# The protein secretion modulator TMED9 drives CNIH4/TGFα/GLI signaling opposing TMED3-WNT-TCF to promote colon cancer metastases

**DOI:** 10.1038/s41388-019-0845-z

**Published:** 2019-06-28

**Authors:** Sonakshi Mishra, Carolina Bernal, Marianna Silvano, Santosh Anand, Ariel Ruiz i Altaba

**Affiliations:** Department of Genetic Medicine and Development, Faculty of Medicine, University of Geneva Medical School, 1 rue Michel Servet, CH1211 Geneva, Switzerland

**Keywords:** Metastasis, Mechanisms of disease

## Abstract

How cells in primary tumors initially become pro-metastatic is not understood. A previous genome-wide RNAi screen uncovered colon cancer metastatic suppressor and WNT promoting functions of TMED3, a member of the p24 ER-to-Golgi protein secretion family. Repression of canonical WNT signaling upon knockdown (kd) of TMED3 might thus be sufficient to drive metastases. However, searching for transcriptional influences on other family members here we find that TMED3 kd leads to enhanced *TMED9*, that TMED9 acts downstream of TMED3 and that *TMED9* kd compromises metastasis. Importantly, TMED9 pro-metastatic function is linked to but distinct from the repression of TMED3-WNT-TCF signaling. Functional rescue of the migratory deficiency of TMED9 kd cells identifies TGFα as a mediator of TMED9 pro-metastatic activity. Moreover, TMED9 kd compromises the biogenesis, and thus function, of TGFα. Analyses in three colon cancer cell types highlight a TMED9-dependent gene set that includes *CNIH4*, a member of the CORNICHON family of TGFα exporters. Our data indicate that *TGFA* and *CNIH4*, which display predictive value for disease-free survival, promote colon cancer cell metastatic behavior, and suggest that TMED9 pro-metastatic function involves the modulation of the secretion of TGFα ligand. Finally, TMED9/TMED3 antagonism impacts WNT-TCF and GLI signaling, where TMED9 primacy over TMED3 leads to the establishment of a positive feedback loop together with CNIH4, TGFα, and GLI1 that enhances metastases. We propose that primary colon cancer cells can transition between two states characterized by secretion-transcription regulatory loops gated by TMED3 and TMED9 that modulate their metastatic proclivities.

## Introduction

The mechanisms that drive the development of metastatic states within primary tumors remain ill defined [1]. These are likely at work in cells with stem cell properties, leading to the appearance of metastasis initiating cells, and at least in colon and breast cancers there is little correlation of the time of metastatic spread with primary tumor size [2, 3]. Genomic analyses in colon or other cancers suggest that metastases reflect the distant expansion of specific cells already present in heterogenous primary tumors without common metastatic-specific driver mutations [4, 5].

Previous work has shown that WNT-TCF signaling in colon cancer is anti-metastatic since its direct repression in grafted human cancer cells enhances metastatic behavior [6, 7]. This anti-metastatic role of WNT-TCF signaling is consistent with the in vivo, unbiased identification of the positive WNT-TCF modulator TMED3 as an endogenous suppressor of distant colon cancer metastases [8].

TMED3 belongs to a family of p24 proteins involved in selecting cargo in COP vesicles in the secretory ER-Golgi network [9]. Given the large diversity of cargo and the existence of only 10 TMED p24 proteins, it is likely that each can affect multiple secretion events in direct and indirect context-dependent manners. Moreover, p24 proteins can exist as monomers or dynamic complexes where one can affect the stability of others [10–13]. They appear to be non-redundant [13, 14] and affect multiple signaling pathways in mammalian cells [15–19]. In flies and mammals, specific TMED proteins control WNT secretion [8, 20–22], and both TMED3 and WNT-TCF signaling act as metastatic suppressors in human colon cancer cells [6–8]. How TMED3 may repress the establishment of pro-metastatic states, however, remains unknown.

## Results

### TMED3 regulates the mRNA levels of other *TMED* family members

**Fig. 1:**
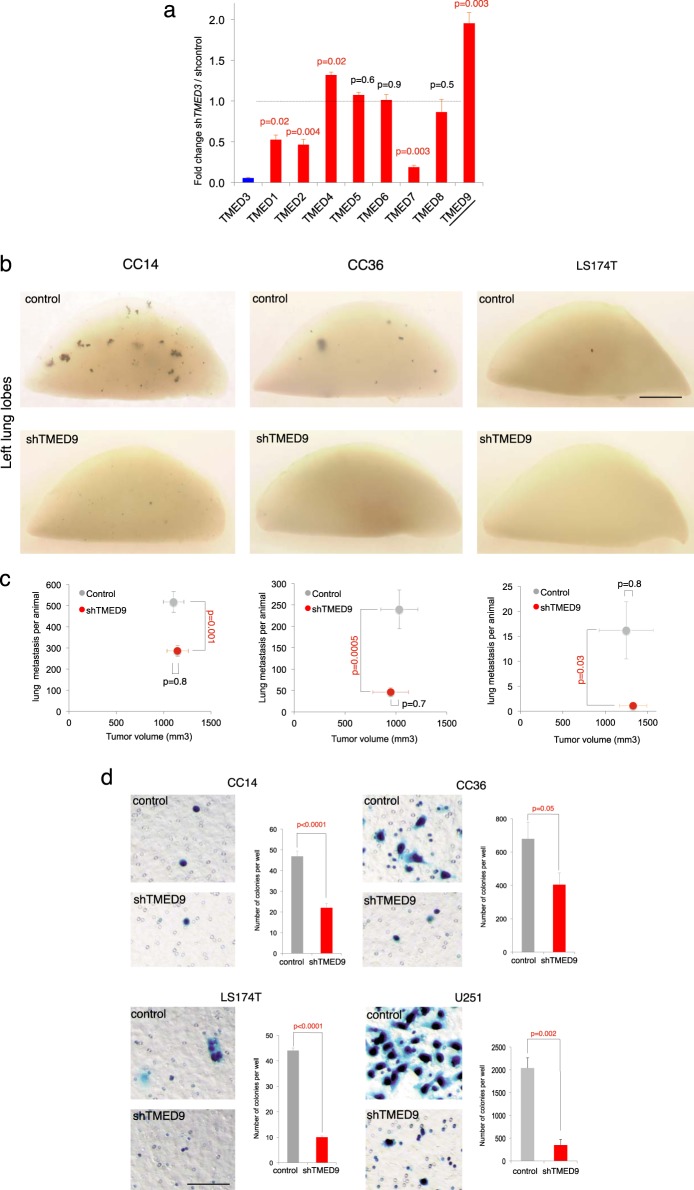
Regulation of the *TMED* family by kd of TMED3 and metastatic phenotypes of cells with kd of TMED9. **a** Histogram of rt-qPCR results for *TMED* mRNA levels in CC14 cells expressing *shTMED3*. Numbers are ratios of normalized CT values of *shTMED3* over control cells. Note that *TMED8* has been suggested to lie outside of the p24 family [9]. *TMED10* was not detected in CC14 cells. **b** Whole views of X-Gal-stained left lung lobes showing metastases in blue as noted. **c** Quantification of the number of *lacZ*^*+*^ lung metastases per animal vs. primary xenograft volume under the different experimental conditions. Each animal carried one tumor per flank. The number of mice involved for CC14 grafts were seven for vector alone control cells and eight for *shTMED9* cells; for CC36 grafts the numbers were seven for control cells and nine for *shTMED9* cells; and for Ls174 grafts they were five for control cells and seven for *shTMED9* cells. **d** Kd of *TMED9* reduces cancer cell migration. Images and quantification of *lacZ*^*+*^ cells (in blue) that have crossed the membrane in transfilter assays for different colon cancer (CC14, CC36, LS174T) and glioblastoma (U251) cells as noted, detected after X-Gal staining cells within the filter. Quantification derives from triplicate experiments with independent batches. In this and all figures, error bars are s.e.m. and *p* values from two-tailed Student's *t* -tests are in red if significant (*p* < 0.05). Scale bar = 0.25 cm for **b** and 80 µm for **d**

To elucidate how blockade of TMED3 promotes pro-metastatic states in primary colon cancer cells, we first investigated if it could affect the expression of other *TMED* family members. Knockdown (kd) of *TMED3* was achieved in CC14 primary human colon cancer cells [23], which are *E*^*1554*^->frameshift APC mutant [7], using a previously validated [8] specific short-hairpin RNA (*shTMED3* with kd of 95%; Fig. 1a). *TMED9* was the only one upregulated more than twofold, whereas several *TMED* genes were downregulated, out of which *TMED7* showed the greatest decrease (Fig. 1a).

### TMED9 is required for distant metastases

Little is known about the TMED family in cancer and, specifically, nothing is known about the possible participation of TMED9 and TMED7 in metastases. This was therefore tested by subcutaneously grafting CC14 cells transduced with lentivectors expressing either *shTMED7* (with kd of 80%) or *shTMED9* (with kd of 90%) and inspecting the lungs of the recipient mice 4 weeks later for distant metastases. We tracked CC14 cells expressing lentivirus-encoded β-galactosidase (CC14^*lacZ*^) in order to detect distant metastases after the X-Gal reaction at single-cell resolution [24]; Fig. 1b, c. Kd of *TMED9*, but not of *TMED7*, resulted in a significant change in the number of metastases with little change in grafted primary tumor volume (Supplemental Fig. 1a, b). *TMED9* kd produced a similar reduction in micro and larger metastases (Fig. 1b, Supplemental Fig. 1c–e). The requirement of TMED9 for distant metastases was recapitulated in primary human colon cancer CC36^*lacZ*^ cells [23] and in the human colon cancer cell line LS174T^*lacZ*^ (Fig. 1b, c, Supplemental Fig. 1d). A second shRNA against *TMED9* with kd of 96% used to validate the initial data yielded a similar result (Supplemental Fig. 1e). Rare liver metastases were also abrogated by kd of *TMED9* (Supplemental Fig. 2).

The metastatic phenotypes were fully recapitulated by the Boyden chamber transfilter assay testing for cancer cell migration [25]; Fig. 1d. Using this assay, TMED9 was shown to be similarly required for the migration of human U251 glioblastoma cells (Fig. 1d), a tumor cell type that readily invades the brain parenchyma [26] used here to test whether TMED9 kd might also affect other tumor types.

As colon cancer metastases derive, at least in part, from CD133^+^ cancer stem cells [27] we quantified their abundance but did not find a difference between parental vs. *shTMED9* pools (5% vs. 5.2% for CC14; 0.14% vs. 0.15% for CC36). This result suggests that the reduction of metastases is not simply due to the loss of CD133^+^ cancer stem cells upon kd of TMED9.

### TMED9 is epistatic to TMED3

**Fig. 2:**
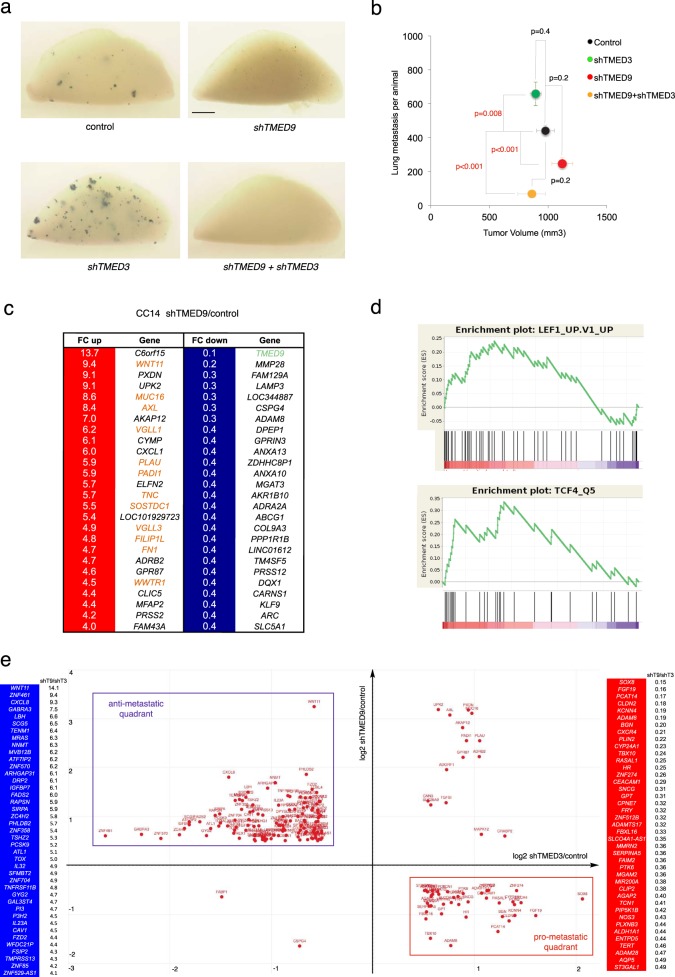
Epistatic analysis and global opposite regulation by TMED9 and TMED3. **a** Images of left lung lobes after X-Gal staining showing metastatic colonies in blue from grafted cells expressing shRNAs as noted. **b** Quantification of the number of lung metastases per animal vs. primary xenograft size (tumor volume). Each animal carried one tumor per flank. The number of engrafted mice involved were eight for control cells, seven for *shTMED3* cells, six for *shTMED9* cells, and eight for *shTMED9* *+* *shTMED3* cells. **c** Heat map of RNAseq data from CC14^*shTMED9*^ vs. CC14^*vectoralone*^ (aka CC14^*control*^) control cells. Genes are ranked by fold change (FC) revealing upregulated (white numbers in red background) and downregulated (white numbers in blue background) genes. Only the topmost genes in each ranked list are shown. Upregulated genes include a significant number of WNT pathway components (their names are in red; see also Supplemental Fig. 3). The lowest FC value corresponds to *TMED9* in green. **d** GSEA enrichment plots of upregulated genes in CC14^*shTMED9*^ vs. CC14^*vectoralone*^ cells showing enrichment of a LEF1 oncogenic signature in human DLD1 colon cancer cells (top panel, http://software.broadinstitute.org/gsea/msigdb/cards/LEF1_UP.V1_UP) and of a TCF binding site signature with binding sites within 4 kb of the transcriptional start site (bottom panel, http://software.broadinstitute.org/gsea/msigdb/geneset_page.jsp?geneSetName = TCF4_Q5). CC14^*shTMED9*^ cells show a positive gene set enrichment in both cases. **e** Comparison of the co-regulated genes by TMED9 and TMED3 from RNAseq of *shTMED9* and *shTMED3* CC14 cells. Values of fold change in mRNA expression levels are plotted in log2 on a two-axis map. Note that the vast majority are found either in the top left (*shTMED9*^*high*^*;shTMED3*^*low*^) putative anti-metastatic or in the bottom right (*shTMED9*^*low*^*;shTMED3*^*high*^) putative pro-metastatic quadrants. Lists of potential anti-metastatic (blue background) and pro-metastatic (red background) genes are given on each side of the graph, ranked by *shTMED9*/*shTMED3* normalized fold change ratios. Scale bar = 0.25 cm for **a**

To establish an order of action of TMED3 vs.TMED9 we performed epistatic analyses using shRNAs to kd each gene alone and in combination in CC14 cells and measuring the number of distant metastases. Whereas *shTMED9* decreased and *shTMED3* increased distant lung metastasis compared with controls (Fig. 2a, b), the simultaneous expression of these two shRNAs yielded an *shTMED9*-like phenotype with a drastic decrease of lung metastases (Fig. 2a, b). The increase in metastases by the repression of TMED3 is thus dependent on TMED9 activity. TMED3-WNT signaling could therefore act as an anti-metastatic brake in part by repressing TMED9. However, as repressing WNT signaling is sufficient to enhance metastases [6–8], these results raised the question of how TMED9 and WNT might interact.

### TMED9 positively regulates genes with migratory/invasive functions and represses WNT signaling

To gain insights into the function of TMED9 we compared the transcriptomes of CC14 cells expressing *shTMED9* (CC14^*shTMED9*^) and control infected sibling (CC14^control^) cells. Inspection of regulated genes (Fig. 2c, Supplemental Fig. 3a) revealed potential mechanisms for the anti-metastatic phenotype of kd of *TMED9*. For instance, genes with enhanced expression included metastatic suppressors (*AKAP12)*, and genes with repressed expression included genes involved in EMT *(MMP28, ADAM8, SNAI3)* and cancer progression *(DPEP1, LAMP3, GSPG4*). Some of these have been correlated with poor prognosis in different cancer types [28, 29]. Kd of *TMED9* did not significantly alter the expression of other *TMED* genes.

Gene ontology enrichment analyses on the resulting RNAseq data revealed global alterations of components of the extracellular space and endoplasmic reticulum (ER) (Supplemental Fig. 3b). Mining public experimental data obtained from human colon cancer cells highlighted an upregulated enrichment of canonical WNT signaling in cells with kd of *TMED9* (LEF1; Gene Set Enrichment Analysis (GSEA); Oncogenic Signatures normalized enrichment score (NES) = 2.05; Fig. 2d top). This was supported by the correlation of highly expressed genes in CC14^*shTMED9*^ cells with those having TCF-binding sites within 4 kb of the start site (GSEA transcription factors; NES = 2.21; Fig. 2d bottom). Indeed, upregulated genes by kd of *TMED9* included a considerable number of WNT signaling components, targets or modifiers in multiple contexts such as *WNT11*, *WNT3*, *MUC16, VGLL1, SOSTDC1*, and *LGR5* (Fig. 2c, Supplemental Fig. 3 [30–32]. *WNT11* has been widely reported to act in the non-canonical migratory/planar polarity/Ca^++^ pathways and repress canonical WNT-TCF signaling [33–35], although it may also act canonically [36]; see below. These results can be consistent with an anti-metastatic role of elevated canonical WNT signaling [6] but less so with a pro-metastatic function of non-canonical WNT signaling observed in other cancers [35, 37, 38].

*TMED9* kd cells also show enhanced levels of *VGLL1*, *VGLL3*, and *WWTR1* (aka *TAZ)* (Fig. 2c, Supplemental Fig. 3a), which encode structural homologs that, like YAP, interact with TEAD transcription factors [39]. Their common increase could implicate HIPPO-TEAD signaling. However, the activity of a TEAD-binding-site->luciferase construct was not upregulated in *shTMED9* as compared with control cells (one- vs. 1.2-fold, *p* > 0.05). These changes could therefore suggest instead a further boost of WNT signaling responses mediated by TAZ/VGLL [32, 40, 41].

Taken together, the data indicate that TMED9 normally represses different aspects of WNT signaling.

### Global opposite gene regulation by TMED9 and TMED3

To address if TMED9 and TMED3 might exert global antagonistic effects we analyzed the transcriptome of cells with kd of *TMED3* and compared it with that of cells with kd of *TMED9*.

Analyses of transcripts altered in *TMED3* kd cells over control levels revealed a group of 63 RNAs enhanced twofold or more that included several genes previously shown to promote pro-metastatic behavior in different cancers, including *SOX8*, *ASCL2, FGF19*, and *CXCR4* (Supplemental Fig. 4). It also revealed a group of 146 transcripts, in addition to *TMED3*, repressed twofold or more that included multiple ZNF zinc finger proteins as well as transcription factor determinants such as *DACH1* (Supplemental Fig. 4), a repressor of colon cancer tumor cell migration and invasion [42].

Comparison of *shTMED3* vs. *shTMED9* RNAseq data yielded 179 transcripts regulated by both (Supplemental Fig. 5). Plotting their expression as fold change over control in a graph with orthogonal axes showed that the great majority of genes (91%) was regulated in an opposite manner (Fig. 2e): 68% in the putative anti-metastatic *shTMED9*^*high*^;*shTMED3*^*low*^ quadrant and 23% in the putative pro-metastatic *shTMED9*^*low*^*;shTMED3*^*high*^ quadrant, versus only 8% in the high:high and 1% in the low:low quadrants.

Top putative pro-metastatic genes listed according to *shTMED9/shTMED3* gene expression ratios (Fig. 2e list in red) included *FGF19, CLDN2*, *KCNN4, ADAM8*, *BGN, PCAT14*, and *CXCR4*, which have been previously linked to pro-migratory, invasive, or metastatic behaviors in different cancers [43–49].

Conversely, putative anti-metastatic genes (Fig. 2e list in blue) included WNT signaling components (*WNT11*, *LGR5*, *FZD2*) and targets *LBH* [50], potential negative modulators of growth, migration, or invasion: *IGFBP7*, *SFMBT2,* and *ARHGAP31* [51–53], as well as *TSHZ2*, a GLI inhibitor [54] (Fig. 2e, Supplemental Fig. 5).

The results show that TMED9 has pro-metastatic function and that it works below TMED3 in this context. Furthermore, they indicate that the antagonistic actions of these two TMED proteins control metastases via the global regulation of multiple genes that participate in the metastatic process. However, what is not known is if both TMED proteins regulate the same or diverse signaling events.

### A common signature of *shTMED9* in different primary colon cancer cells reveals genes encoding ER-Golgi network proteins

To begin to address TMED9 actions in more detail we sought to delineate a conserved gene expression signature with which to track, albeit indirectly, TMED9 activity. Thus, to complement the findings on CC14 cells we determined the transcriptomes of CC36^*control*^ and CC36^*shTMED9*^ cells since both CC36 and CC14 are primary colon cancer uncloned cell populations [23] and both respond to *shTMED9* similarly (Fig. 1). Both have been previously determined to harbor active WNT signaling and produce enhanced metastases in response to WNT blockade by dnTCF [6, 7].

Enrichment analyses of the CC36^*shTMED9*^ vs. CC36^*control*^ transcriptomes did not highlight changes in WNT signaling (Supplemental Fig. 6), which may be owing to the different nature of the cells: CC36 cells do not form epithelial colonies as CC14 cells do. Instead, they display a mesenchymal phenotype with enhanced > 50-fold migration as compared with CC14 [23] and aspects of WNT signaling or its responses are already downregulated. For example, comparison of normalized baseline rt-qPCR ct gene expression values revealed generally higher expression of WNT signaling components/targets in CC14 vs. CC36 cells: *WNT3* 4.4-fold, *WNT3a* 3.2-fold, *AXIN2* 2.3-fold, *WNT11* 1.2-fold, *LGR5* > 500-fold.

**Fig. 3:**
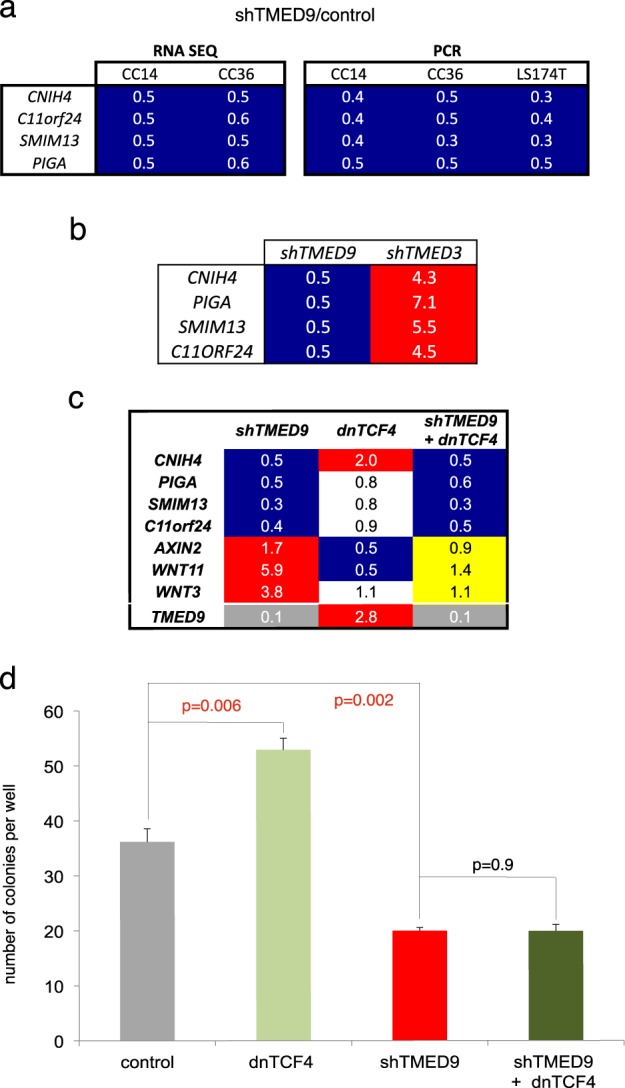
A conserved TMED9 kd signature and interaction with WNT-TCF signaling. **a** Heat map of shared repressed genes from deep sequencing (RNA seq) and rt-qPCR (PCR) values in three different colon cancer cells as shown. These four genes constitute a *shTMED9* signature. **b** Heat map of rt-qPCR results testing for the expression of the four-gene TMED9-dependent signature in CC14^*shTMED9*^ and CC14^*shTMED3*^ cells. Note the opposite regulation by these two TMED factors. PCR was used here as the expression values of this signature in the *shTMED3* RNA seq data did not pass the FDR cutoff. **c** Heat map of rt-qPCR normalized ratios for the three noted conditions (each over control) for the selected genes. The rescue of the expression of WNT pathway genes (yellow background), but not those of the *TMED9* signature, is afforded by combined TMED9 kd and repression of WNT-TCF signaling by dnTCF4. Kd levels of *TMED9* are in gray background. **d** Quantification of cells crossing the membrane in transfilter experiments with repressed WNT-TCF signaling (dnTCF4) and/or *shTMED9* cells as noted. DnTCF4 does not rescue (enhance) the compromised migration of cells with kd of *TMED9*

Global comparison of gene expression changes shared between CC36^*shTMED9*^ and CC14^*shTMED9*^ cells over their respective controls thus allowed us to search for common effects of *TMED9* kd beyond WNT signaling (Supplemental Fig. 6). Using FDR < 0.05 and fold change of two yielded 9 repressed and 11 induced candidates. These were then re-tested by rt-qPCR in three independent CC14^*shTMED9*^ and CC36^*shTMED9*^ batches as well as in LS174T^*shTMED9*^ cells vs. their respective controls. Four repressed genes were thus identified encoding CNIH4, Phosphatidylinositol glycan anchor biosynthesis class A (PIGA), the integral small membrane protein SMIM13, and the single-pass type I integral membrane protein C11orf24 (Fig. 3a). All four proteins are localized in the secretory network as are the p24 proteins [55, 56]. The CORNICHON family displays phylogenetically conserved TGFα export function [57–59] and CNIH4 has also been involved in the secretion of G-protein coupled receptors [60] and defined as a cargo adaptor [61]. PIGA is the first enzyme required for the production of the GPI moiety of all GPI-linked membrane proteins [62], which require p24 function for export [63, 64]. This conserved four-gene *TMED9*-dependent signature highlights secretion-transcription regulatory mechanisms and we use it henceforth to track TMED9 activity. Indeed, this signature also responded in opposite ways to kd of *TMED3* vs. kd of *TMED9* (Fig. 3b), in agreement with the global analyses shown above.

### TMED9 pro-metastatic activity is linked to but separate from WNT signaling inhibition

Having determined a conserved TMED9-dependent signature, we addressed whether the actions of TMED9 may be solely effected via regulation of WNT signaling, perhaps in an opposite manner to TMED3. This was first addressed by directly repressing WNT signaling responses cell autonomously through the use of a dominant negative form of TCF4 (dnTCF), both in the absence and in the presence of kd of *TMED9*. Lentivector transduction of dnTCF alone resulted in the downregulation of *AXIN2*, a canonical WNT responsive gene, and of *WNT11*, encoding a WNT ligand, as expected. Moreover, it was also able to rescue their upregulation, and that of *WNT3*, by *shTMED9* (Fig. 3c), indicating that TMED9 and WNT signaling may balance each other.

Direct blockade of TCF function, however, was unable to rescue the repression of *CNIH4, PIGA, SMIM13*, or *c11orf24* driven by *shTMED9* (Fig. 3c), arguing for a separate effect of TMED9.

In functional assays, dnTCF induced a higher number of migrating CC14 cells in transfilter assays, consistent with the anti-metastatic function of WNT-TCF signaling [6]. However, it was unable to rescue the migration deficiency imposed by *shTMED9* (Fig. 3d), paralleling the gene expression results above. We interpret these results to indicate that the pro-metastatic activity of TMED9 is not simply due to its blockade of canonical WNT function, arguing for the existence of additional TMED9-dependent pro-metastatic signals.

### The TMED9 and TMED3 responsive gene *CNIH4* is required for metastasis

To validate the significance of the TMED9-regulated signature and to begin to investigate possible pro-metastatic events downstream of TMED9, we chose to test the function of CNIH4, which belongs to a family of evolutionarily conserved TGFα exporters [57, 58].

**Fig. 4:**
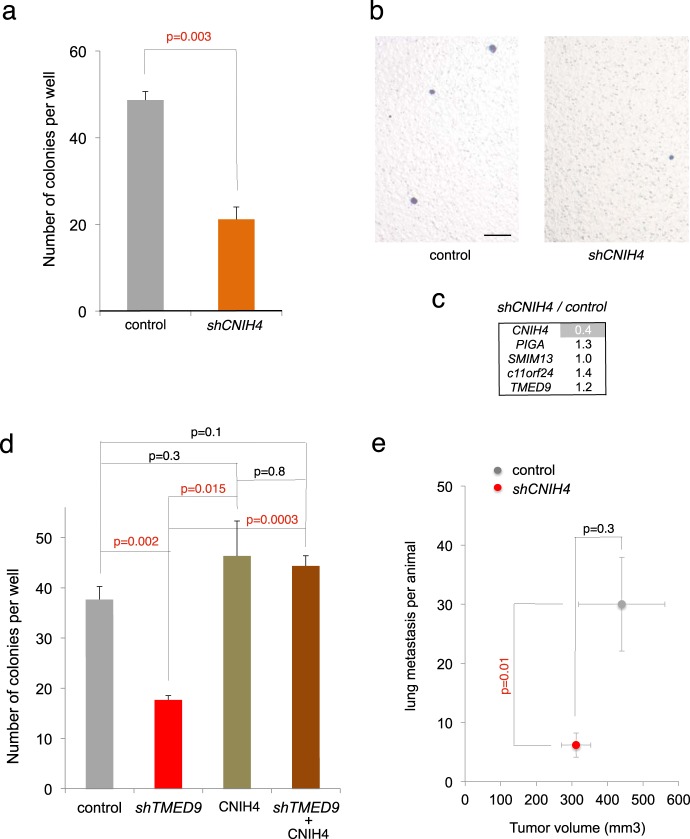
CORNICHON 4 is required for metastases. **a**, **b** Effects of kd of *CNIH4* in CC14 cells on transfilter migration (**a** quantification; **b** representative images). **c** Heat map of the expression levels of the selected genes in *shCNIH4* cells over the values in control cells, after normalization. The kd of *CNIH4* is shown in gray background. **d** Enhanced levels of CNIH4 using a cDNA vector in transient transfections rescues the migratory deficiency of *shTMED9* cells in transwell assays. On its own enhanced CNIH4 does not increase the basal levels. **e** In vivo quantification of CC14^*lacZ*^ lung metastases per animal under the different experimental conditions noted. *CNIH4* kd reduced the number of metastases without altering tumor volume. Each animal carried one tumor per flank. *n* = 5 grafts for control vector alone cells and *n* = 6 grafts for *shCNIH4* cells. Scale bar = 80 µm for **b**

Use of a lentivector-encoded shRNA reducing *CNIH4* mRNA levels by 95% resulted in a 60% reduction of transfilter CC14 cell migration (Fig. 4a, b). Similar results were obtained with a second shRNA with kd of 90% (Supplemental Fig. 7a). Kd of *CNIH4* did not affect the expression of *TMED9* or the other TMED9-dependent signature genes (Fig. 4c). Conversely, enhanced CNIH4 levels using a cDNA in transiently transfected cells rescued the transwell migratory deficiency of *shTMED9*, although it did not enhance the basal level of control cells (Fig. 4d). Enhanced CNIH4 levels did not affect the expression of the other TMED9 signature genes (not shown). Importantly, and in line with the in vitro data, engrafted CC14^*shCNIH4*^ cells produced tumors with reduced numbers of distant metastases without significantly altering primary tumor size, all as compared with CC14^control^ cells (Fig. 4e).

These results link *CNIH4*, a TMED9- and TMED3-regulated gene, to the positive control of metastases.

### TGFα rescues the migratory deficiency of colon cancer cells with compromised TMED9 function

Given the nature of TMED9 as a secretory cargo selector and the requirement of TMED9 and CNIH4 for metastases shown above, TGFα as well as other ligands previously implicated in tumor progression or metastases—SONIC HEDGEHOG (SHH) [6], FGF1 [65], FGF19 [44], and TRAIL [66]—were tested for their ability to rescue the decreased migratory phenotype of *shTMED9* cells.

**Fig. 5:**
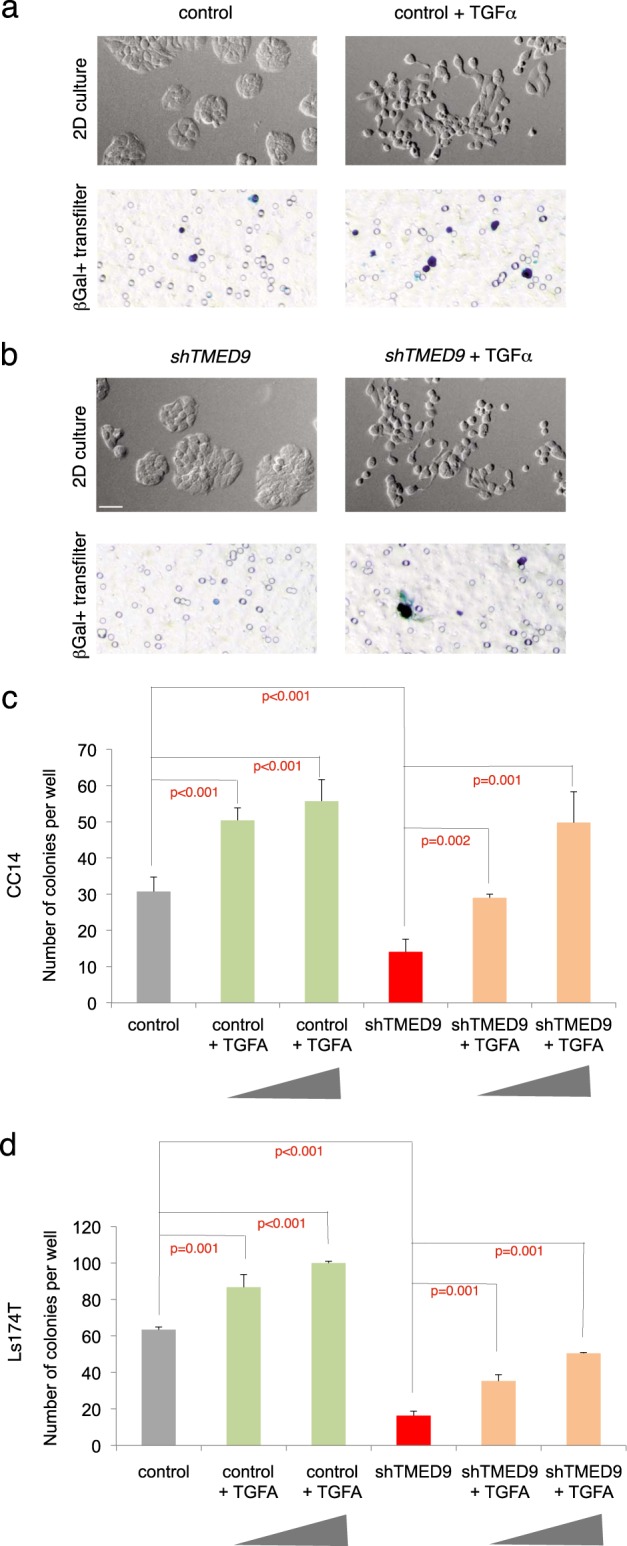
TGFα rescues *shTMED9* phenotypes. **a**, **b** Inverted Nomarski images of 2D colonies in vitro (top panels with gray background) and transmitted light images of X-GAL-stained cells that crossed the filter in transfilter assays (bottom panels with white background) showing the effects of TGFα ligand on *lacZ*^*+*^ CC14^control^ **a** and *lacZ*^*+*^ CC14^*shTMED9*^ **b** cells. TGFα ligand induces EMT-like disaggregation, cell dispersion, and migration, bypassing shTMED9-induced migratory deficiencies. **c**, **d** Quantification of transfilter results in CC14 **c** and LS174T **d** cells. See Supplemental Fig. 10 for CC36 data. Concentrations of TGFα (gray triangles) were 10 and 25 ng/ml

This was performed using the transfilter system to ascertain their effects specifically on human tumor cells: cells were pre-treated with ligands for 48 h and plated with and without their continued presence. The only tested molecule able to rescue the transfilter migration deficiency of cells with kd of *TMED9* under either experimental strategy was TGFα, which also induced the EMT-like disaggregation of CC14 epithelial colonies (Fig. 5a–c, Supplemental Fig. 8, 9). TGFα effects were reproduced in LS174T and CC36 cells, suggesting their widespread effects on human colon cancer cells (Fig. 5d, Supplemental Fig. 10). TGFα did not rescue the transfilter deficiency of glioblastoma U251 cells with compromised *TMED9* function, used here as outlier controls (Supplemental Fig. 10).

The colon cancer cells responding to TGFα ligand were determined by DNA sequencing to harbor oncogenic mutations (KRAS^G13D^ and BRAF^wt^ in CC36; KRAS^wt^ and BRAF^V600E^ in CC14, KRAS^G12D^, and BRAF^wt^ in LS174T) downstream of the TGFα receptor EGFR. These cells might thus have been suspected a priori to be insensitive to signaling by EGFR ligands given the action of RAS–RAF signaling downstream of TGFα−EGFR function. Interestingly, EGF treatment was unable to mimic the effects of TGFα (Supplemental Fig. 8), suggesting the existence of EGFR ligand selectivity [67] for pro-metastatic function.

### Blockade of the TGFα receptor EGFR and kd of *TGFA* decreases colon cancer cell migration and metastases

To investigate in more detail the mechanisms involved in controlling pro-metastatic states we have focused henceforth on patient-derived, primary CC14 cells as these display a clear epithelial morphology in vitro and in xenografts, mimicking local, and early primary colon cancers [6].

**Fig. 6:**
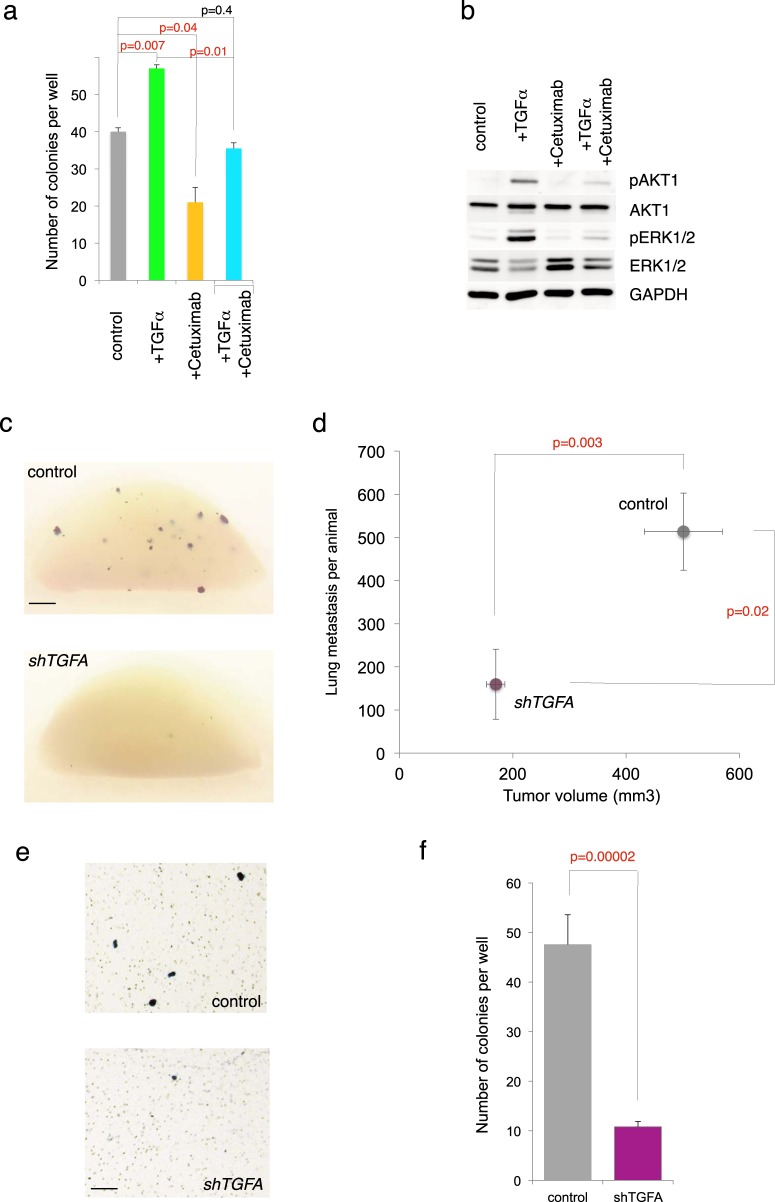
Inhibition of TGFα–EGFR signaling represses cell migration and metastases. **a** Quantification of transfilter experiments showing the modulation of the number of migrating/invading cells by TGFα ligand and by blocking EGFR activity, the TGFα receptor, with Cetuximab. **b** Analysis of the phosphorylation of AKT1 and ERK1/2 by Western blotting in sibling cells to those used in **a** 5’ after treatments. Total AKT1, total ERK1/2 and GAPDH were used as controls. **c**, **d** Representative images **c** and quantification **d** of *lacZ*^*+*^ lung metastases in mice carrying CC14^*lacZ/*control^ or CC14^*lacZ/shTGFA*^ xenografts. The number of metastases is compared with the size of xenografts in **d**. Each animal carried a single subcutaneous flank tumor. *n* = 8 vector alone control and *n* = 6 *shTGFA* engrafted mice. **e**, **f** Images **e** and quantification **f** of transfilter experiments with CC14^control^ and CC14^*lacZ/shTGFA*^ cells showing the strong reduction in migrating cells after *TGFA* kd. Scale bar = 0.25 cm for **c**, 80 µm for **e**

Events downstream of TGFα ligand action were probed by blocking the activation of its receptor, EGFR, with the monoclonal antibody Cetuximab [68]. Blocking EGFR resulted in a 50% reduction in the number of migrating cells in transfilter assays and abrogated the increase afforded by TGFα treatment when cells were incubated with both TGFα and Cetuximab (Fig. 6a). As expected, Cetuximab treatment decreased endogenous events downstream of EGFR in these cells as highlighted by the acute inhibition of p-ERK and AKT levels by 50% and 20%, respectively (Fig. 6b). Blocking EGFR thus yields the expected phenotype in cell migration predicted from the analyses of TGFα gain-of-function described above.

To directly test if endogenous TGFα participates in metastases, we independently used two shRNAs reducing its mRNA levels by 90%. CC14^*shTGFA*^ cells engrafted into NSG mice produced smaller tumors and had fewer metastases as compared with CC14^control^ cells (Fig. 6c, d).

In order to discern a direct effect on the metastatic process rather than as a possible secondary effect owing to reduced tumor size, *shTGFA* cells were tested in transfilter experiments: CC14^*shTGFA*^ produced an 80% reduction in transfilter migration as compared with controls (Fig. 6e, f). A second shRNA with kd of 80% reduced the number of cells crossing the filter by 60% (Supplemental Fig. 7b). These results show that TGFα and its receptor EGFR are required for experimental colon cancer metastases.

### Compromised TGFα biogenesis in cells with kd of TMED9

**Fig. 7:**
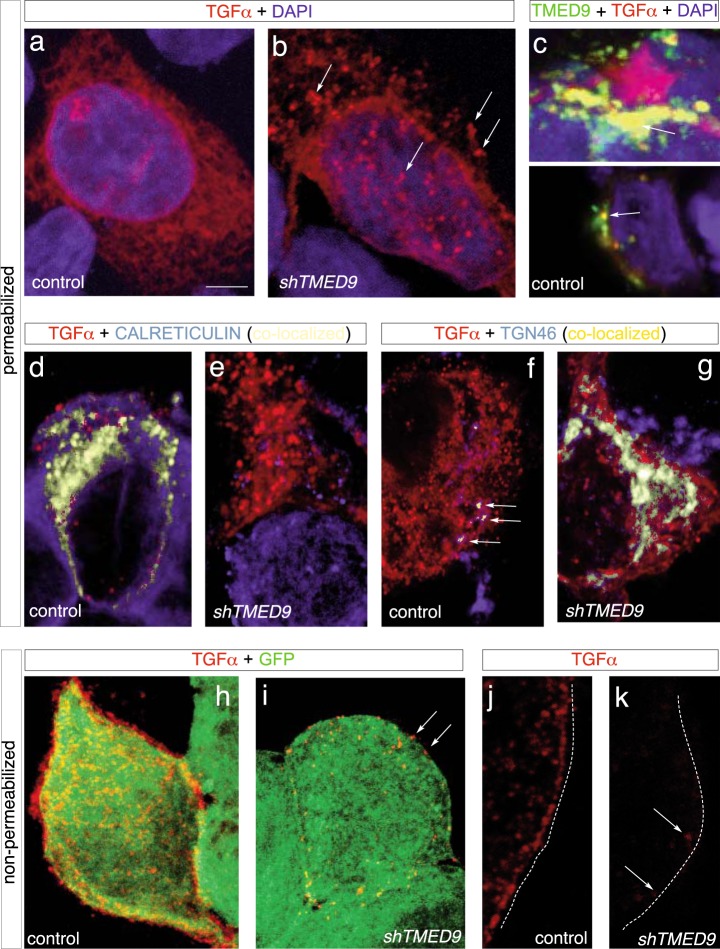
Partial colocalization of TMED9 and TGFα and abnormal biogenesis of TGFα in cells with compromised TMED9 function. **a–g** Immunolabeling in CC14 permeabilized cells. **a**, **b** Intracellular distribution of TGFα (red) in control (CC14^*vectoralone*^) **a** and *shTMED9* (CC14^*shTMED9*^) cells **b**. The normal distribution of TGFα in the secretory machinery near the nucleus (blue after DAPI staining) is disrupted in cells expressing *shTMED9* in which large aggregates appear (arrows in **b**) in > 30% of cells analyzed. Images show maximal projections of confocal 0.4 µm z-stacks. **c** Colocalization of TGFα and TMED9 in co-transfected CC14 cells using HA-TGFα and Myc-TMED9 (green). Two single confocal image sections of 0.4 µm are shown highlighting the partial (yellow) overlap near the DAPI^+^ (blue) nuclei. **d**, **e** Predominant localization of TGFα in the CALRETICULIN^+^ ER in control **d** as compared with *shTMED9* **e** cells. Here and in **f**, **g** colocalization (arrows) is shown in single confocal images of 0.4 µm of thickness using ImageJ to detect overlap (highlighted in greenish white). Two independent examples are shown in the upper and lower panels. **f**, **g** TGFα accumulates abnormally in the TGN46^+^ Golgi in *shTMED9* **g** as compared with control **f** cells, in which only partial localization is observed (arrows in **f**). **h–k** Immunolabeling in non-permeabilized CC14 cells showing the localization of transfected TGFα (in red) in control **h**, **j** or *shTMED9* **i, k** cells expressing **h**, **i** or not **j**, **k** GFP in their cytoplasms. Arrows point to residual membrane expression in **i**, **k**. **h**, **i** show maximal projections of confocal 0.4 µm z-stacks where the red signal in the center of the cell in **h** is on the top membrane of the cell. **j**, **k** show single confocal 0.4 µm sections. Scale bar = 4 µm for all panels except 1 µm for (**c** upper panel, **j**, **k)**

To investigate how TMED9 might control TGFα function, we investigated the localization of the latter in cells with normal or compromised TMED9 function. Epitope tagged TGFα [69] was immunolocalized in permeabilized cells as a smooth layer with fine puncta over the secretory network as expected ([69]; Fig. 7a). In cells with compromised *TMED9* this pattern was commonly replaced by large aggregates (Fig. 7b). Double immunolabeling against co-transfected HA-tagged-TGFα and MYC-tagged-TMED9 revealed partial colocalization in structures close to the nucleus (Fig. 7c upper and lower panels): TGFα localized largely to the Calreticulin^+^ ER and less to the TGN46^+^ Golgi (Fig. 7d, f). Conversely, in *shTMED9* cells TGFα strongly localized within the Golgi and weakly in the ER (Fig. 7e, g). As TGFα was not localized in EEA1^+^ endosomes or LAMP1^+^ lysosomes (Supplemental Fig. 11) it appeared retained in the Golgi in cells with kd of *TMED9*. Co-immunoprecipitation analyses failed to yield positive results, likely due to the small amounts of ligand present.

The possibility that TGFα was retained intracelullarly in cells with compromised TMED9 was tested by immunolocalizing membrane-bound tagged TGFα precursors in non-permeabilized cells using maximal projection of z-stacked confocal images to visualize the entire cell surface. TGFα precursors were normally localized in the membrane of control GFP^+^ cells but their abundance was greatly diminished in cells with kd of *TMED9*, with the strongest effect yielding little TGFα signal (Fig. 7h–k). Quantification of immunopositive dots reveled a 10-fold decrease in *shTMED9* vs. control cells (Fig. 7h, i). As membrane localization of TGFα is required for membrane signaling as well as for the cleavage to produce secreted ligands, its biogenesis and overall function is compromised in cells lacking normal TMED9 function.

To complement the immunofluorescence results, we analyzed overall cell surface TGFα levels using cell surface biotinylation and pull-down of biotinylated proteins, followed by Western blotting in CC14^vectoralone^ vs. CC14^*shTMED9*^ cells expressing tagged TGFα. Using this method we observed that the 36 kD membrane form of TGFα was only detected in the membrane fraction, as expected, but also that it was reduced twofold in *shTMED9* cell surface fraction as compared with control cells. As controls, EGFR, was enriched 2.5-fold in the membrane and the cytosolic protein HSP70 inversely enriched twofold in the non-membrane fraction (Supplemental Fig. S12a).

In addition, enzyme-linked immunosorbent assay (ELISA) immunodetection of secreted TGFα present in the condition media of CC14^*vectoralone*^ and CC14^*shTMED9*^ cells showed twofold reduction, on average, in the latter (Supplemental Fig. 12b).

### The levels of *TGFA* and *CNIH4* predict disease outcome

Taken together with previous data [8], the results presented above indicate that TMED3 and TMED9 act as gatekeepers for protein secretion events that lead to the regulation of TGFα and WNT signaling, establishing antagonistic regulatory modes that control metastatic states within primary tumors. To analyze the possibility that their expression predict disease outcome in patients we have correlated their expression levels with disease-free survival using public cohorts.

**Fig. 8:**
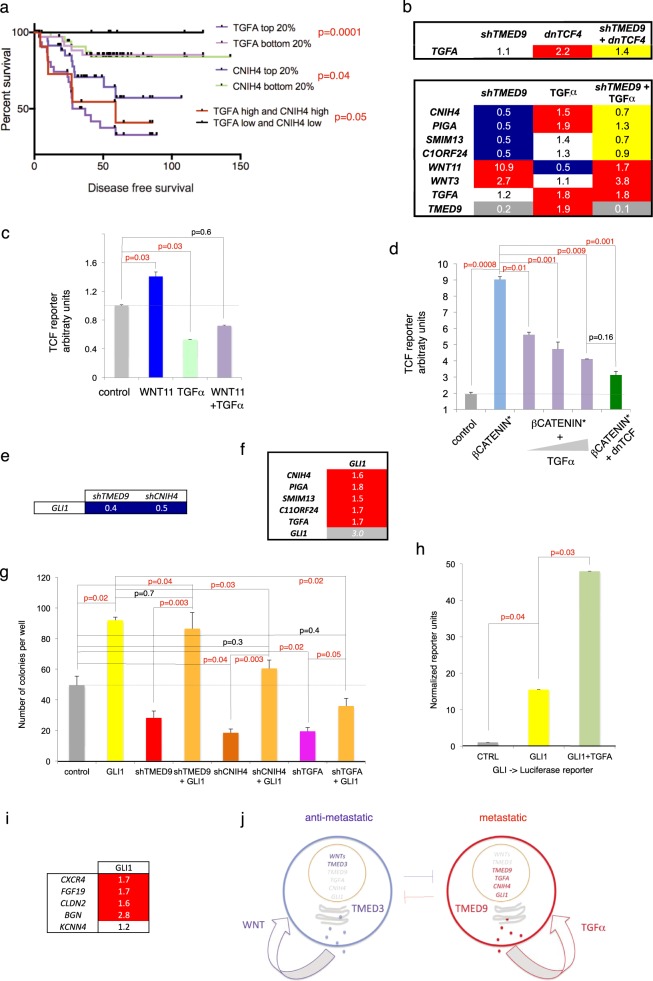
The levels of *CNIH4* and *TGFA* predict disease outcome, interactions with WNT and GLI signaling and a two-state model for the regulation of metastatic states. **a** Kaplan–Meier survival curves showing the relationship between the expression levels of *TGFA* and *CNIH4* and disease-free survival in colon cancer noted in months. The results of high vs. low cohorts, alone or together, are shown compared with the inverse cohorts. *P* values are shown next to the legend of each combination. **b** Heat map of normalized rt-qPCR ct value ratios of experimental over control cells as indicated. Top: reversion of the enhancement of the levels of *TGFA* mRNA induced by dnTCF4 by simultaneous kd of TMED9 (*shTMED9*). Bottom: reversion of the repression of the TMED9-dependent gene signature, but not of WNT pathway components, by TGFα. In both cases, rescued values are highlighted in yellow. **c**, **d** Histograms of TCF-> uciferase reporter activity in CC14 cells as noted after normalization with internal Renilla and mutant binding site (FOP) controls. **c** Whereas Wnt induces (blue histogram) and TGFα represses (green histogram) endogenous TCF reporter activity, together they show mutual compensation (light violet histogram). **d** Similarly, increasing concentrations of transfected *TGFA* (100, 250, and 500 ng) abolished the increase in TCF-> Luciferase reporter activity afforded by exogenous N’-mutant activated βCATENIN (βCATENIN*), leading to repression equal to that seen after co-expression of dnTCF. **e**, **f** Heat maps showing the downregulation of *GLI1* by *shTMED9* and *shCNIH4* **e** and the upregulation of the TMED9-dependent signature by GLI1 **f**. **g** Quantification of transfilter experiments results showing the ability of transfected GLI1 to rescue the migration defects of *shTMED9, shCNIH4,* and *shTGFA* cells and increase the number of control cells crossing the filter. Note that full GLI1 activity is hampered by kd of *CNIH4* or *TGFA*. **h** Histograms of changes in GLI Luciferase reporter activity demonstrating the ability of TGFα ligand treatment (10 ng/ml for 48 h) to superinduce the activity of GLI1. Values have been normalized by internal Renilla and by mutant GLI binding site controls. **i** Heat map of the common induction of 4/5 tested *shTMED9*^*low*^*;shTMED3*^*high*^ pro-metastatic genes by GLI1. **j** Diagrams of proposed anti- and pro-metastatic states determined by antagonistic TMED3- and TMED9-gated signaling loops. Ligands may act in autocrine and paracrine modes. The two states are self-sustaining and mutually repressive. Pro-metastatic states may arise from the inhibition of WNT signaling or reprogramming. Inversely, WNT signaling from the metastatic niche stroma may reverse a pro-metastatic state towards a local mode of growth to expand metastatic lesions locally. See text for additional details. All values in heat maps are normalized ratios over control CC14 cells **b**, **e**, **f**, **i**

*TMED9* quintiles showed a trend where top expressors showed poorer outcomes, but these did not reach significance of *p* = 0.05 (Supplemental Fig. 13), much as reported for *TMED3* [8]. These results may reflect the fact that each TMED protein controls the secretion of different cargoes and the activity of several signaling pathways [70], and that TMED proteins are detected at all colon cancer stages (https://www.proteinatlas.org/search/TMED). We therefore analyzed the correlation of the TMED regulated genes *TGFA* and *CNIH4* with disease-free progression. High *TGFA* [71] or *CNIH4* quintiles showed a strong correlation with disease outcome and the combination of both *TGFA*^*high*^, *CNIH4*^*high*^ vs. *TGFA*^*low*^, *CNIH4l*^*ow*^ was also positively correlated with poor outcome (Fig. 8a).

### TGFα shows a mutually antagonizing relationship with WNT-TCF signaling and rescues the gene signature repressed by kd of *TMED9*

Having determined the requirement of TGFα, we asked how TGFα and the WNT pathway may affect each other since we show that TMED9 represses *WNT11* and other signaling components and responses (Fig. 2) and TCF represses (dnTCF enhances) *TMED9*.

We found that dnTCF enhanced *TGFA* levels, suggesting the coordinate repression of *TMED9* and *TGFA* by WNT-TCF. Inversely, TGFα ligand treatment repressed *WNT11* (Fig. 8b) and *TGFA* kd-induced WNT11 2.3-fold. Moreover, TGFα compensated the boost of *WNT11* induced by *shTMED9* (Fig. 8b). This shows the ability of TGFα to revert a number of TMED9 kd phenotypes (see also Fig. 5) and its ability to repress *WNT11*. *TGFA* mRNA levels were also enhanced by TGFα treatment (Fig. 8b), pointing to its auto-induction as in keratinocytes [72].

We then asked if TGFα could antagonize WNT signaling using a TCF luciferase reporter: WNT11 enhanced and TGFα repressed endogenous TCF activity, whereas together they yielded an intermediate phenotype, arguing that they antagonize each other’s effects (Fig. 8c). Indeed, such antagonism was also revealed through the exogenous activation of WNT signaling using active N’-mutated βCATENIN (βCATENIN*): increasing doses of TGFα co-transfected with βCATENIN* led to the repression of the activating effect of the latter on a TCF Luciferase reporter to levels similar to those detected after the repression by dnTCF (Fig. 8d).

At last, whereas kd of *TGFA* did not alter the TMED9-dependent gene signature (not shown), treatment with TGFα ligand enhanced the expression of *CNIH4*, *PIGA,* and *TMED9* itself and, notably, it rescued the downregulation of the TMED9-dependent signature by *shTMED9* (Fig. 8b). This result, together with the requirement of *TGFA* shown above (Fig. 6), indicates that TGFα mediates many, but not all, aspects of TMED9 function, that it shows a mutually antagonizing relationship with WNT-TCF signaling, and that TGFα signaling can be self-reinforcing (Fig. 8j).

### GLI1 rescues the kd phenotypes of TMED9, CNIH4 and TGFα, establishing a pro-mestastatic regulatory loop

Previous work has identified GLI activity in colon cancer as pro-metastatic acting in part to antagonize WNT-TCF signaling [6, 23]. As this parallels the roles of TMED9 and TGFα described above, we addressed the possibility that GLI1 could mediate aspects of TMED9-TGFα activity, as we found that both *shTMED9* and *shCNIH4* decreased *GLI1* expression levels (Fig. 8e), and GLI1 was able to enhance the expression of *TMED9* by 40% as well as the TMED9-dependent signature, thus mimicking *shTMED3* (Fig. 8f).

Functionally, cells expressing lentivector-encoded shRNAs against *GLI1* were not viable enough to perform assays (not shown). Nevertheless, we were able to determine that GLI1 rescued the transfilter migratory deficiency imposed by kd of *TMED9*, *TGFA, or CNIH4*: Exogenous GLI1 (yielding a 3–5-fold increase in mRNA levels over controls) induced a twofold enhancement in the number of CC14 cells crossing the filter and, importantly, rescued the phenotype of *shTMED9*, *shCNIH4* and *shTGFA* to control levels (Fig. 8g). Inversely, the positive effect of GLI1 was unopposed by *shTMED9* but decreased in cells with kd of *CNIH4* or *TGFA* (Fig. 8g). This indicates that whereas GLI1 rescues all three phenotypes, full GLI1 function requires endogenous CNIH4 and TGFA activities.

The results then prompted us to investigate the possibility of a mutual dependency between *GLI1* and *TGFA*. We found that GLI1 was able to enhance the mRNA levels of *TGFA* although TGFα did not affect *GLI1* expression (Fig. 8f and not shown). However, TGFα did enhance GLI1 activity threefold, measured with a GLI-binding-site-> luciferase reporter (Fig. 8h).

Taken together the results are consistent with the idea that pro-metastatic actions of TMED9, CNIH4, and TGFα establish a positive regulatory loop with GLI1. Further support for this possibility derived from the finding that 5/6 *shTMED9*^*low*^*;shTMED3*^*high*^ pro-metastatic genes (Fig. 2e) tested were GLI1-responsive (Fig. 8i). Globally, the idea that protein secretion-transcription loops operate to determine metastatic proclivities in primary tumor cells is also suggested by the finding that *TMED9*, *CNIH4*, as well as *TGFA* [73], harbor high-confidence consensus GLI-binding sites within 5 Kb upstream of the transcriptional start site, whereas *TMED3* harbors TCF-binding sites instead (Supplemental Fig. 14).

## Discussion

A critical step in metastasis is the decision of cells within primary tumors to acquire a pro-metastatic state, which when realized will allow cells to migrate, disperse, and eventually colonize distant sites. How such states are initially established is not understood, in part owing to the difficulty in deciphering context-dependent actions and the complexity of cross-talk among signaling pathways. Here, we provide evidence that a critical mechanism in the positive control of pro-metastatic states, and the resulting distant metastases, in human colon cancer cells is regulated protein secretion, as highlighted by the function of the protein secretion cargo selector TMED9.

Little is known on the function of individual TMED proteins in normal human cells or in cancer. TMED9 (aka p24alpha2 or p25) has been shown to form complexes with other TMED proteins ([10, 12]; https://string-db.org) but its role in metastasis remained largely unexplored [74, 75]. In the present study, we show that *TMED9* is transcriptionally negatively regulated by TMED3, and that TMED9 has a pro-metastatic function and works in an epistatic manner in relation to TMED3. We find that TMED9 and TMED3 activities balance each other to determine metastatic outcomes and control in opposite manners a global gene cohort that includes multiple factors implicated in the regulation of metastases.

A previous unbiased in vivo RNAi screen revealed TMED3 as a positive modulator of WNT signaling [8] and blocking WNT-TCF signaling with dnTCF has been shown to promote metastases from multiple primary colon cancer cells [6, 7]. Consistently, here we show that WNT-TCF signaling represses *TMED9* and in turn TMED9 represses WNT-TCF pathway components and responses. However, we show that although the metastatic outcome of repression of WNT signaling requires TMED9, the pro-metastatic function of TMED9 is separate from repression of WNT signaling. Therefore, TMED9 not only exhibits a mutually repressive interaction with TMED3-WNT but must also positively regulate pro-metastatic signals.

Defining TMED9-regulated genes and searching for factors able to rescue TMED9 kd-induced functional deficiencies allow us to uncover TGFα, a high-affinity ligand for EGFR [69, 76], as a mediator of pro-metastatic TMED9 function: TGFα ligand, rescues the phenotypes of kd of TMED9 and provokes the dispersion of colon cancer epithelial colonies, as in other cancer cells [77]. Although endogenous cargoes remain to be identified for TMED9, four lines of evidence suggest that TMED9 promotes TGFα activity: (i) We find that cancer cells with compromised TMED9 function have reduced membrane and secreted TGFα levels, indicating that TGFα requires TMED9 for normal biogenesis and thus function; (ii) TMED9 is present in TGFα-containing and in COPII secretory vesicles [78, 79]; (iii) TMED proteins associate with GRASP55/65 (GORASP1/2 [80]), which binds precursor and membrane forms of TGFα [81]; (iv) We show that CNIH4, which encodes a member of the CORNICHON family of evolutionarily conserved TGFα exporters, is required for metastases and is regulated by TMED9 activity, and like TMED9, CNIH4 is also found in COPII vesicles [79]. TMED9, however, is likely to regulate signals other than TGFα. Moreover, it remains unclear if CNIH4 directly regulates TGFα secretion given that cells with overexpressed CNIH4 via lentivector transduction were not viable (not shown) to perform ligand localization analyses, and CNIH4 has been also shown to export G-protein coupled receptors [60].

The results presented above, the presence of secretion-transcription regulatory loops suggested by the control of the expression of *CNIH4* and *TGFA* by TMED9 and TMED3, the positive regulation of *GLI1* by TMED9 and CNIH4, the requirement of normal TGFα activity, as well as by the ability of GLI1 to rescue the kd phenotypes of *TMED9, CNIH4,* and *TGFA*, together with previous findings on the roles of WNT and GLI signaling in colon cancer metastases [6–8], allow us to propose the existence of two antagonistic, self-sustaining loops that regulate the pro-metastatic states of colon cancer cells (Fig. 8j).

1- A basal pro-tumorigenic but anti-metastatic state is afforded by fully activated WNT signaling, usually via APC mutation, as well as by TMED3-mediated secretion of WNT ligands, as these are required for full WNT-TCF pathway activation even in the presence of pathway-activating mutations [82]. We posit that full WNT-TCF activity is self-reinforcing and expands the tumor. Moreover, it keeps tumor growth local through repression of pro-metastatic TMED9-TGFα signaling via its downregulation of TMED9, CNIH4, TGFA, and GLI1.

2- The self-sustaining local, anti-metastatic WNT-TCF loop in colon cancer cells is proposed to rebalance in favor of pro-metastatic TMED9, CNIH4, TGFα, GLI signaling during the metastatic transition, which involves WNT signaling repression (e.g., *WNT* ligand and *TMED3* downregulation) and the enhancement of the expression levels and function of *TMED9*, *CNIH4*, *TGFA,* and *GLI1* (Fig. 8j). This may happen in single or small numbers of cells and is consistent with both the wide distribution of βCATENIN, with high levels commonly due to APC mutation, (https://www.proteinatlas.org/ENSG00000168036-CTNNB1/pathology/tissue/colorectal+cancer#img) and the heterogenous expression of high levels of TGFα (https://www.proteinatlas.org/ENSG00000163235-TGFA/pathology/tissue/colorectal+cancer#img) in human colon cancers.

How the stable TMED3-WNT-TCF autocrine loop is derailed is unclear. This might involve the upregulation of WNT inhibitors (e.g. *DKK1* and *SFRP1*) as observed in metastatic vs. non-metastatic colon cancer patients [6], but also oncogene-mediated increases in GLI function [24, 83] and cell-intrinsic metastatic reprogramming [84]. Enhanced and self-sustaining TMED9-gated ligand signaling in turn is predicted to further downregulate the TMED3-gated WNT-TCF loop and promote pro-metastatic states.

In this model, GLI1 can act both upstream and downstream of TMED9, CNIH4, and TGFα. Whereas direct GLI activation by oncogenic signals may help break the WNT–TMED3 loop (see above), its downstream regulation by TMED9-TGFα signaling is consistent with the ability of peptide growth factors, as well as oncogenic downstream mediators such as RAS, MEK, and AKT (reviewed in [83, 85], to boost positive GLI function and enhance metastases [6, 23]. Moreover, TMED3/TMED9 co-regulated genes include genes shown to be GLI-responsive such as *CLDN2, CXCR4,* and *FGF19* [73], although whether the combined action of TGFα and GLI1 can drive a gene set distinct from that driven by GLI1 alone, as shown for EGF [73], remains to be determined. Enhanced GLI1 activity can thus establish a regulatory loop with TGFα (this work) and decrease WNT-TCF activity [6].

An additional mechanism to reinforce the balance in favor of TMED9-TGFα-GLI signaling may rely on NAKED2 (NKD2), as post-Golgi TGFα (after TMED9 and CNIH4 function) requires NKD2 for vesicular export [86, 87]. Mammalian NKD2 also targets the essential WNT pathway component Disheveled for degradation thus further inhibiting WNT signaling [88].

We note that in normal development and regeneration, these signaling pathways and components are very sensitive to dosage, working as morphogens. Small changes in the activities of WNT and TGFα in cancer cells might therefore have important metastatic outcomes. In this sense, the antagonism of the TMED9-TGFα and TMED3-WNT loops may not act solely cell autonomously, with the final balance and outcome being influenced by both signaling within tumor cells and from the surrounding stroma: the cancer cell-intrinsic and the stroma-to-cancer cell pro-metastatic functions of TGFα may therefore coexist and cooperate ([89, 90]; this work).

The balancing act of opposing TMED3-WNT-TCF and TMED9-TGFα signaling loops in the determination of pro-metastatic fates may be akin to those found in development and regeneration. WNT and TGFα participate in the architecture of normal and regenerating crypt-villus axes of the intestine and are expressed at opposite ends: canonical WNT ligands are made in the bottom part of the crypt and promote crypt stem cell self-renewal [91]. In contrast, TGFα activity is enriched in the villus and promotes cellular differentiation and migration towards the tip, also affecting cells in the crypt [92–95]. Epithelial cells in villi loose responsiveness to TGFα upon differentiation and are eventually shed into the lumen [96]. Metastatic cells may re-differentiate and repress TGFα signaling, regaining an epithelial morphology and re-entering a local mode of growth to establish metastatic colonies. In metastatic colon cancer, such a reversal might be accomplished by re-establishing the primacy of WNT signaling for local expansion through non-cell autonomous niche-mediated mechanisms [97].

More generally, we suggest that TMED protein secretion cargo selectors may have context-dependent roles in the regulation of migratory/invasive/metastatic behaviors in different cancer types. For example, TMED3 can promote anti-metastatic WNT signaling in colon cancer cells [8], whereas it has been reported to promote IL11 signaling and tumor progression in liver cancer cells [70]. Thus, whereas *TMED9* levels per se are not significantly correlated with disease-free survival, the levels of mediators and markers we identify - *CNIH4* and *TGF*α- predict colon cancer outcome.

We propose that antagonistic secretion-transcription loops gated by TMED9 and TMED3 represent a key modulatory network for the control of the number of colon cancer metastatic cells.

## Materials and methods

### Cells, lentivectors, and plasmids

Patient-derived CC14 and CC36 primary colon cancer cells [23] were maintained as early passage attached cultures in DMEM-F12 medium supplemented with 5% fetal bovine serum (FBS) and penicillin/streptomycin. LS174T human colon cancer cells (ATCC) were maintained in MEM medium plus 10% FBS and PS. All cells were mycoplasma-free as tested by PCR. shRNAs against *TMED2*, *TMED7,* and *TMED9* generated by oligo cloning in the LV-CTH vector used alone as control [98], were: *TMED2* (5’CTCGGGCTATTTCGTTAGCAT3’ and 5’CATGGATGGAACATACAAAT3’); *TMED7* (5’GCCTGTGTTTCAATTCACGAA3’ and 5’CGAAGCTCTGAAGTCTGTCAT3’); *TMED9* (5’GCTGCTAAAGACAAGTTGAGT, and 5’ GAAGTGCTTTATTGAGGAGAT3’). pGIPZ-s*hTMED3* [8] used the pGIPZ-vector alone as control. Other constructs used were: *shTGFA* (Sigma TRCN0000006373 and TRCN0000364608), *TMED9*-*MYC-DDK* (Origene RC200652), *TGFA-GFP* (Origene RG218141), *shCNIH4* (Sigma TRCN0000183590 and TRCN0000184650), CNIH4-Myc-DDK (Origene RC200050), pCS2-Myc‐tagged human GLI1 [99], *dnTCF4*, TOP, and FOP luciferase reporters were kind gifts of H. Clevers (Utrecht University), 8XGTIIC-luciferase (synthetic TEAD luciferase reporter (Addgene 34615), GBS, GBS mutant, and *N’∆βCATENIN* (used as in 99,6). FLAG and HA-tagged *TGFA* was a kind gift of Dr. Coffey (Vanderbilt University [100],). Production of lentiviral particles was as described [24]. shRNA lentivectors for the same gene were used separately.

### Tumor engraftment and distant metastases

Colon cancer cells expressing the *lacZ* tracer were transduced with additional lentivectors at a MOI of 2–3. Cells infected with lentivectors carrying GFP tracers, were sorted by fluorescence-activated cell sorting (BD biosciences ARIA III) 72 h afterwards and replated. 48 h after sorting, cells were trypsinized, counted. For pLKO.1 lentivectors, 48 h after infection puromycin (5 µg/ml) selection was performed and cells were plated for experiment after further 48 h. In total, 5 × 10^5^ cells were re-suspended in 100 µl of Hanks' Balanced Salt Solution (HBSS) solution for subcutaneous injections per site into the flanks of 8–12 week immunocompromised NSG (NOD-scid IL2rg^null^) mice purchased from Charles River. Xenografts of *shCNIH4* cells and controls were performed in NUDE mice purchased from Janvier Laboratories. Mice were allocated to control or experimental samples randomly from the cage but not blindly. All mice were female and were cared for and kept at the SPF University of Geneva’s Animal Facility. Sample sizes and power were determined with G*Power3. Xenografted subcutaneous tumor growth was monitored by measuring its volume with a caliper. Mice were sacrificed before tumors reached legal limits (according to Swiss (Office Cantonal de Vétérinaire de Genève) animal care standards) and xenografts and lungs harvested following approved protocols (GE/77/17). Rare (< 1 in 30) outliers with tumor sizes above or below two standard deviations from the average were excluded from the study. Lungs were fixed in fresh 4% paraformaldehyde (PFA) for 4 h and stained with X-Gal for 6 h to identify β-Galactosidase^+^ cells. Stained lungs were washed with phosphate-buffered saline (PBS) 4–5 times and positive cells/colonies were counted under a dissecting microscope as described in [24]. *LacZ*^*+*^ cells were counted on both sides of all lung lobes per mouse.

### Transfilter assays

CC14^*lacZ*^, CC36^*lacZ*^, or LS174T^*lacZ*^ cells transduced with different lentivectors were plated as adherent cultures to reach 70–75% confluence the next day. Cells were washed twice with HBSS and replenished with 0.5% FBS containing medium (serum-deprived medium) for 16 hours. They were then trypsinized to obtain single cells. 2 × 10^5^ cells were re-suspended in 100 µl of serum-deprived medium and placed on the upper chamber of pre-hydrated transwells (6.5 mm diameter, 8.0 µm pore size, Corning #3422). In total, 650 µl of medium supplemented with 10%FBS was placed in the lower chamber of the transwell insert to serve as attractant. CC14^*lacZ*^ and LS174T^*lacZ*^ cells were incubated for 72 h, whereas CC36^*lacZ*^ needed only 24 h. Subsequently, the contents of the upper chambers were carefully aspirated and the membranes washed with PBS, fixed for 5 min in 4% PFA, re-washed with PBS thrice and stained with X-Gal. The upper part of the membrane was subsequently cleaned gently with a cotton bud to remove any remaining cells on the surface of the filter. X-Gal stained cells were counted and photographed with an inverted optical microscope. All assays were performed at least twice in triplicates. In the case of GLI1 transfilter assay, DNA transfection with 2 µg of GLI1 plasmid was performed with 10^6^ cells in P60 dishes using Lipofectamine LTX or with Amaxa Nucleofection reagent. After 48 h, the cells were plated for transfilter assays as described above. All assays were performed at least twice in triplicates.

### Ligand and cetuximab treatments

Cells at 50% confluence were washed twice with HBSS and fed with 0.5% FBS medium containing ligands for 24–48 h: TGFα (Abcam ab9587); SHH (Genescript Z03067, C24II); TRAIL (Abcam ab9960); FGF1 (Sinobiological 10013-HNAE); EGF (Genescript Z00333-10), FGF19 (Sigma SRP4542). Cells were then harvested and plated for transwell assays with or without continued presence of ligands. Duplicates were used for real-time quantitative PCR (rt-qPCR) and western blots.

CC14 cells were treated with 2 µg/ml Cetuximab (Biovision, #A1047), TGFα (10 ng/ml) or with both at the same time for 72 h and plated for transfilter assays. For western blot analyses, cells were plated at the density of 2 × 10^5^ cells in 10 cm plates and treated the day after with Cetuximab for 72 h in 0.1% serum condition medium followed by 5 min treatment with TGFα at 10 ng/ml.

### Rt-qPCR

RNA extraction, cDNA synthesis, rt-qPCR, primer sequences and data analyses were performed by standard techniques as described [6, 23, 24, 86]. Other primers were as follows. All 5–3’: *CNIH4*: Fw:TCAACTTACCTGTTGCCACTTG, Rev: TCTGTTGGATCAAACACTCCCA; *PIGA*: Fw: GGTATATGACCGGGTATCAGTGG, Rev: GCAAAGATGTAGCCTGTTACTGG; *SMIM13*: Fw: AGTGGGTGAAAATTCCCGCT, Rev: CCCTGGTAAACACTCAGCCC; *C11orf24*: Fw: TCCAACGATCCACGCAACTTT, Rev: CATGGTTACATCCTCAGACGTTT; *WNT11*: Fw: CAGGATCCCAAGCCAATAAA, Rev: TATCGGGTCTTGAGGTCAGC; *WNT3*: Fw: AGGGCACCTCCACCATTTG, Rev: GACACTAACACGCCGAAGTCA; *TGFA*: Fw: AGGTCCGAAAACACTGTGAGT, Rev: AGCAAGCGGTTCTTCCCTTC; *TMED9*: Fw: GCGCTCTACTTTCACATCGG, Rev: CACCTCCACAAACATGCCAA.

### RNA sequencing and transciptome analyses

Oligo dT-selected mRNAs isolated from three independent experiments for CC14^*vectoralone*^ vs. CC14^*shTMED9*^ and for CC36^*vectoralone*^ vs. CC36^*shTMED9*^ cells were subjected to deep sequencing on the Illumina platform of the University of Geneva’s Genomic Facility. The sequencing was performed with 50 or 100 bp reads and ~30 million of reads per sample. Differentially expressed genes were identified using edgeR [101] software and the P values adjusted with a 1% false detection rate (FDR) using the Benjamini–Hochberg correction unless otherwise noted. Analyses of regulated genes were performed with Enrichr (http://amp.pharm.mssm.edu/Enrichr/), GSEA (http://software.broadinstitute.org/gsea/index.jsp) and GOrilla (http://cbl-gorilla.cs.technion.ac.il/) for enrichment analyses. RNA sequencing data are deposited to GEO (accession number GSE125282).

### Western blotting

Cells were lysed in radioimmunoprecipitation assay buffer buffer containing a protease and phosphatase inhibitor cocktail (Sigma P-2714, Thermoscientific 1862495). Whole-cell lysates were fractioned on SDS-polyacrylamide gels, blotted to nitrocellulose membranes and incubated overnight with the following antibodies: p-AKT ser473 (dilution 1:1000; 9271 Cell Signaling), p-ERK1-2 (dilution 1: 1000; sc-7383 (E-4) SantaCruz), AKT (dilution1: 1000; 9272 Cell Signaling), GAPDH 1:2000 (2118 Cell Signaling), EGFR 1:1000 (D38B1 Cell Signaling), HSP70 1:1000 (sc-24 SantaCruz), or HA-Tag (Cell Signaling 2367; 1:1000) followed by incubation with HRP-conjugated secondary antibodies (Promega). For all Cell Signaling primary antibodies, 5% BSA (bovine serum albumin) with TBST (Tris-buffered saline with 0.1% Tween 20) was used as blocking reagent. Immunoreactive bands were visualized by enhanced chemoluminescence (Amersham, GE Healthcare). Densitometry calculations used ImageJ software.

### Cell surface protein isolation

In total, 2 × 10^6^ of each CC14 control and *shTMED9* cells were plated in p100 dishes and transfected 2 days after with 24 µg of HA-tagged *TGFA* plasmid. After 24 hours of transfection, cell surface protein isolation was performed using a cell surface protein isolation kit (ab206998 Abcam) following the manufacturer’s instructions. In brief, cells were first labeled with Sulfo-NHS-SS-Biotin, cells were then lysed and the labeled cell surface proteins isolated using streptavidin beads. Cell surface proteins were finally eluted with 0.1 m DTT and analyzed by western blotting with the antibodies of interest.

### TGFα ELISA assay of CC14-conditioned medium

For conditioned media preparation: 1 × 10^6^ of each CC14 control and *shTMED9* cells were seeded in p60 dishes with complete medium. The day after cells were washed twice with PBS and once with serum-free medium, and covered with 5 ml of serum-free medium. After 48 hours of incubation media were collected, centrifuged and filtered. This conditioned media was immediately used to measure the concentration of TGFα using the Human TGFα ELISA kit (MBS700091 MyBioSource). The optical densities of standards and of our duplicate samples were measured at 450 nm using a Victor3 1420 plate reader.

### Immunofluorescence

Cells were seeded over glass coverslip in 24-wells plates and plasmid transfected the day after with Lipofectamine-2000. The following day cells were fixed with fresh 4% PFA pH8.0, permeabilized and blocked with PBS containing 0.1% Triton X-100 and 10% heat-inactivated normal goat serum (PBSTH). Primary antibodies were incubated overnight at 4 °C at dilutions recommended by the providers in PBSTH. After washing in PBS, secondary fluorescently tagged antibodies were incubated for 1 h at room temperature in PBSTH. For non-permeabilized immunofluorescence with anti-HA antibodies the protocol of Briley et al. [100] was followed with the exception of the replacement of BSA for 10% heat-inactivated goat serum. The efficiencies of transfection and the total numbers of HA-positive cells in each experiment were determined per condition. Images were taken with an LSM700 confocal microscope (0.4 µm slices) at the University of Geneva’s Imaging Facility and processed using FIJI software. Primary antibodies used were anti-: HA-Tag (Cell Signaling 2367; 1/100), TGN46 (ABCAM ab50595, 1/200), cMYC (Santa Cruz Biotechnology sc-40; 1/100), CALRETICULIN (ABCAM ab2907;1/75), EEA1 (Cell Signaling No. 3288; 1/100), and LAMP1 (Cell Signaling No. 9091; 1/200).

### Luciferase assays

Cells were first plated in 24-well plates at a density of 50,000 cells per well and transfected with Lipofectamine the day after either with 500 ng of wild type- (TOP) or mutant (FOP) TCF, GBS, GBS mutant, and 8XGTIIC, binding site luciferase reporters (e.g., 6). In addition, cells were transfected with *N’∆β-CATENIN*, *WNT11-V5* [102] and/or *TGFA* (TGFα-gfp-tagged RG218141, Origene) plasmids for 24 h.

### Data mining for Kaplan–Meier disease outcome plots and binding site predictions

Mining of data were performed using the GSE17538 set containing 238 tumor samples. The clinical follow-up including disease-free survival and event of death for all 238 patients are available in www.ncbi.nlm.nih.gov/geo/query/acc.cgi?acc=GSE17538 [103, 104]. The probes were as mentioned in [84]. or as follows; TMED9- 208757_at, TMED2- 214658_at, TMED7- 214658_at, TMED1- 203679_at, TGFA- 205016_at, CNIH4- 228437_at. *p* values were calculated and survival Kaplan–Meier plots were made with PRISM GRAPHPAD program. For multiple gene analyses, 50% cohorts were independently used for each probe and common samples (e.g., high or low for the genes tested) kept in the multiple cohort.

For each gene, fasta sequence of 5 kb upstream from the transcription start site and 5′-UTR region was obtained from UCSC table browser on the hg19 human genome. The fasta sequences thus obtained were searched for the presence of binding motifs using the program FIMO [105]. FIMO [v4.10.0] was run with default parameters. The position weight matrices (PWMs) for the transcription factors were obtained from the JASPAR database [106]. The following PWMs were searched for binding sites GLI: M01042_GLI1, MA0734.1_GLI2; TCF: MA0523.1_TCF7L2, MA0830.1_TCF4

## Supplementary information

SUPPLEMENTAL INFORMATION
