# Supplementary material for: The protein secretion modulator TMED9 drives CNIH4/TGFα/GLI signaling opposing TMED3-WNT-TCF to promote colon cancer metastases
Source: Oncogene. 2019 Jun 28;38(29):5817–37. doi: 10.1038/s41388-019-0845-z (PMC6755966; doi:10.1038/s41388-019-0845-z)
Supplement: Supplementary file 1 — SUPPLEMENTAL INFORMATION [file 41388_2019_845_MOESM1_ESM.pdf]

Supplemental Information

TMED9 modulates TGFα signaling opposing TMED3-WNT to promote colon cancer metastases

Sonakshi Mishra*[1], Carolina Bernal*[1], Marianna Silvano*[1],

Santosh Anand[1] and Ariel Ruiz i Altaba[+1]

[1]Dept. of Genetic Medicine and Development, Faculty of Medicine, University of Geneva Medical School, 1 rue Miquel Servet, CH1211 Geneva, Switzerland

*Shared first authorship

[+]Corresponding author

Running title: TMED9-gated signaling determines metastatic fates

Supplemental Figures

Supplemental Figure 1. Effects of kd of *TMED7* and distribution of distant metastases per size from *shTMED7* or *shTMED9* grafts.

a) RNA interference of *TMED7* in primary colon cancer CC14 cells ($CC14^{shTMED7}$) did not produce significant changes in tumor volume or the number of distant lung metastases as compared with those derived from $CC14^{vectoralone}$ (control) cells.

b) The distribution of metastases per size was also unaltered in mice bearing $CC14^{shTMED7}$ vc. $CC14^{vectoralone}$ (control) xenografts. Metastasis sizes were divided into three groups in the histograms: larger than 100 cells (in blue), 10 cells or smaller (gray) and in between these two groups (red).

c,d) Distribution of *lacZ*$^{+}$ lung metastases quantified by size in $CC14^{shTMED9}$ (c) and $CC36^{shTMED9}$ (d) cells. All three metastatic size categories were reduced with the largest change being observed in micrometastases (gray).

e) Effects of a second independent shRNA against *TMED9* in CC14 cells showing a large decrease in metastases and, in this case, a smaller decrease in tumor size.

In this and all Supplemental figures error bars represent s.e.m. and P values derive from 2-tailed Student's T-tests.

Supplemental Figure 2. Rare liver metastases from Ls174T xenografts are abolished upon kd of *TMED9*.

a,b) Quantification (a) and representative images (b) of the number of metastases per liver (a). The arrow points to a βGAL^{+} stained liver metastasis.

Scale bar= 0.1cm for (b).

Supplemental Figure 3. Global gene expression changes induced by kd of *TMED9*.

a) Changes in gene expression in $CC14^{shTMED9}$ vs. $CC14^{vectoralone}$ (control) cells by RNA sequencing were ranked by fold change and those above or equal to 2-fold and those below or equal to 0.55 are included. The top genes in each case are shown in Fig. 2c.

b) GOrilla enrichment analyses of Cellular Components using the transcriptomic data of $CC14^{shTMED9}$ vs. $CC14^{vectoralone}$ (control) cells. Note the heat map for FDR-corrected P values and the GO term numbers associated with each highlighted category.

Supplemental Figure 4. Global changes in gene expression in TMED3 kd vs. control cells.

Gene expression list ranked by fold changed (FC) in $CC14^{shTMED3}$ vs. control $CC14^{\text{vectoralone}}$ cells. The results of RNAseq performed in cells expressing a specific shRNA against *TMED3* were filtered by FDR and ranked by FC. The list shows genes induced by 2-fold or more (red background) and those repressed by 2-fold or more (blue background). *TMED3* is highlighted in gray.

Supplemental Figure 5. Complementary list of genes co-regulated in opposite manner by *shTMED9* and *shTMED3*.

The left and central columns list all genes with positive *shTMED9/shTMED3* expression value ratios pertaining to the anti-metastatic quadrant in blue background of Fig. 2e (in which only the top ranked genes are shown). The genes are ranked by fold change. The right column show the genes in *shTMED9*/*shTMED3* high:high (top) and low:low (bottom) quadrants of Fig. 2e.

Supplemental Figure 6. Global comparative heat map of TMED9 kd changes in CC14 and CC36 colon cancer cells.

The heat map shows changes in mRNA levels in CC14^{shTMED9} vs. CC14^{vectoralone} and CC36^{shTMED9} vs. CC36^{vectoralone} cells in triplicates. The blue to red scale is shown above. Values were selected by p value <0.05 only. Four general categories are shown and separated by horizontal green lines. *TMED9* is highlighted by a red arrow towards the top.

Supplemental Figure 7. Effects of second shRNAs against *CNIH4* or *TGFA* on transfilter cell migration.

a, b) Histograms of the quantification of the inhibition of migration of control CC14 cells and those expressing a second shRNA against *CNIH4 (shCNIH4-2)* (a) or *TGFA (shTGFA-2)* (b) in transfilter assays.

Supplemental Figure 8. Effects of pretreatment with EGF, TRAIL, FGF1, FGF19 and SHH on control and TMED9 kd cells.

Histograms show the quantification of CC14^{vectoralone} (control) and CC14^{shTMED9} cells migrating/invading cells in transfilter experiments. Cells were pretreated for 48h with ligands as noted at different concentrations (assessed from literature) and plated without added ligands. None of the treatments shown here rescued the migratory deficiencies of *TMED9* kd cells.

All amounts noted in the figure are per ml.

Supplemental Figure 9. Effects of sustained treatment with TGFα, TRAIL, FGF1 and SHH on control and TMED9 kd cells.

Histograms of the quantification of migrating/invading CC14^{vectoralone} (control) and CC14^{shTMED9} cells in transfilter experiments as in Supplemental Fig. 8. In this case,

cells were continuously treated with ligands as noted before *as well as* during the transfilter experiment. Only TGFα rescued the migratory deficiency of *shTMED9* cells.

All amounts noted in the figure are per ml.

Supplemental Figure 10. Rescue of *shTMED9-induced* migratory deficiencies by TGFα in primary CC36 colon cancer cells but not in U251 glioblastoma cells.

a) Histogram of the quantification of migrating/invading CC36 cells in transfilter experiments. Primary colon cancer CC36 cells were pretreated with TGFα before plating them in the well and challenged to move across the filter. The results in CC36 mimic those obtained with CC14 (see Fig. 5c,d).

b) Histogram of the quantification of migrating/invading U251 cells in transfilter experiments. The cell line U251 was used as an outlier to test the potential generality of TGFα effects on *shTMED9*-expressing cells. Cells were pretreated with TGFα before plating them in the microwells.

All amounts noted in the figure are per ml.

Supplemental Figure 11. Lack of co-localization of TGFα with early endosomes or lysosomes in control or TMED9 kd cells.

Immunolabeling of control or $CC14^{shTMED9}$ cells for the early endosome marker EEA1 (top), or the lysosome marker LAMP1 (bottom), together with tagged transfected TGFα as noted. Nuclei were counterstained with DAPI. Sporadic overlap (in yellow) could be seen with EEA1. However, inspection of individual confocal sections confirmed lack of significant co-localization.

All panels show maximal projections of confocal z-stacks of 0.4μm slices.

Scale bar = 4μm for all panels.

Supplemental Figure 12. Western blots of the pull-down of membrane proteins and ELISA detection of secreted TGFα from CC14^{vectoralone} control and CC14^{shTMED9} cells.

a) Western blots of the same cell extracts showing the decreased membrane localization of the 36KD form TGFα, representing the mature membrane form (99) in CC14 cells with TMED9 kd versus control cells. As controls, EGFR localization was enriched in the membrane and the cytoplasmic HSP70 protein was enriched in the non-membrane fraction, both as expected. Cells were biotinylated and membrane proteins bound with streptavidin beads before total extraction and pull down. Note that Western blotting with anti-tag antibodies of HA-TGFα reveals also a major 25 KD form and an unspecific band of higher molecular weight (*, see ref. 99). The upper anti-TGFα blot is a longer exposure of the part noted in the lower one.

b) Conditioned media (CM) from CC14^{vectoralone} (gray square) and CC14^{shTMED9} (red square) cells were analyzed by Elisa for the detection of secreted TGFα. The red line represent the trendline of standard curve points, built by plotting on the y-axis the O.D. values for each standard and on the x-axis their concentration (pg/ml).

Supplemental Figure 13. Kaplan-Meier curve of *TMED9* mRNA levels versus disease-free survival.

Analyses of high versus low quintiles for *TMED9* showed a trend correlation, without reaching significance, for high *TMED9* levels and poor disease outcome noted in months. Upper and lower tertiles and quartiles also did not reach significance (not shown).

Supplemental Figure 14. Prediction of GLI and TCF binding sites in the regulatory regions of the noted genes.

Genomic sequences of 0 to -5Kb upstream of the transcriptional start site (TSS) were scanned for the presence of consensus GLI or TCF binding sites. The position of the motif in the + or – strand is given, as well as its sequence and FDR as a measure of confidence.

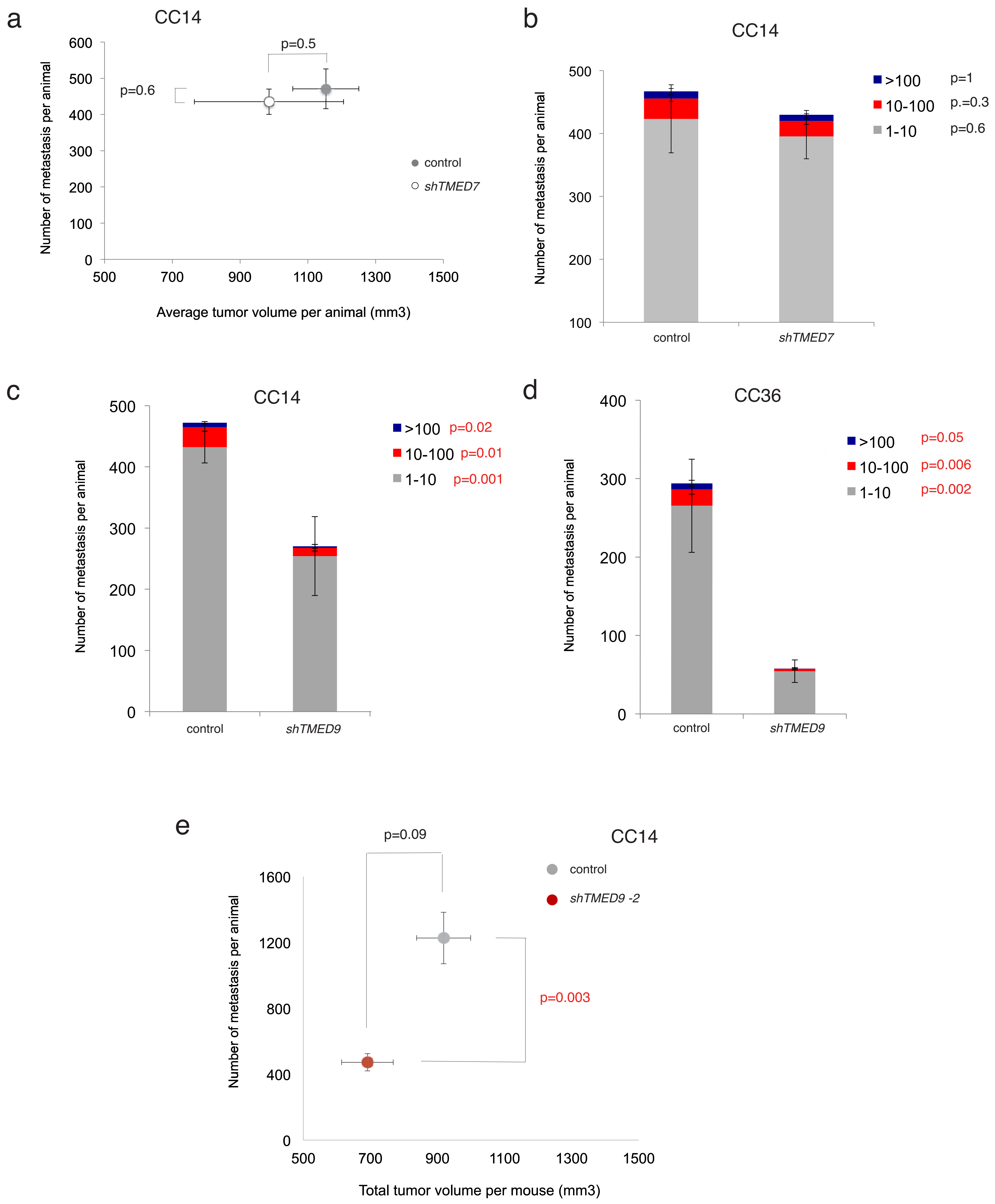

Mishra et al Fig. S1

a

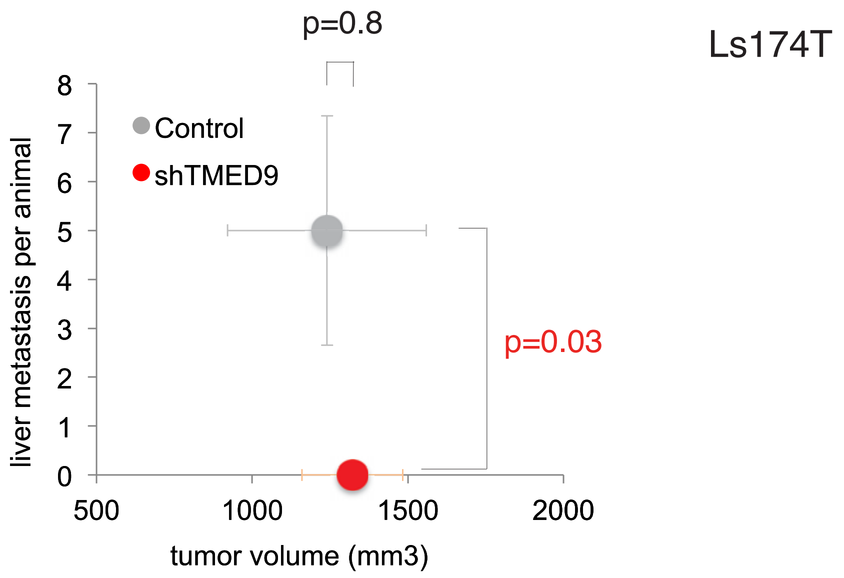

b

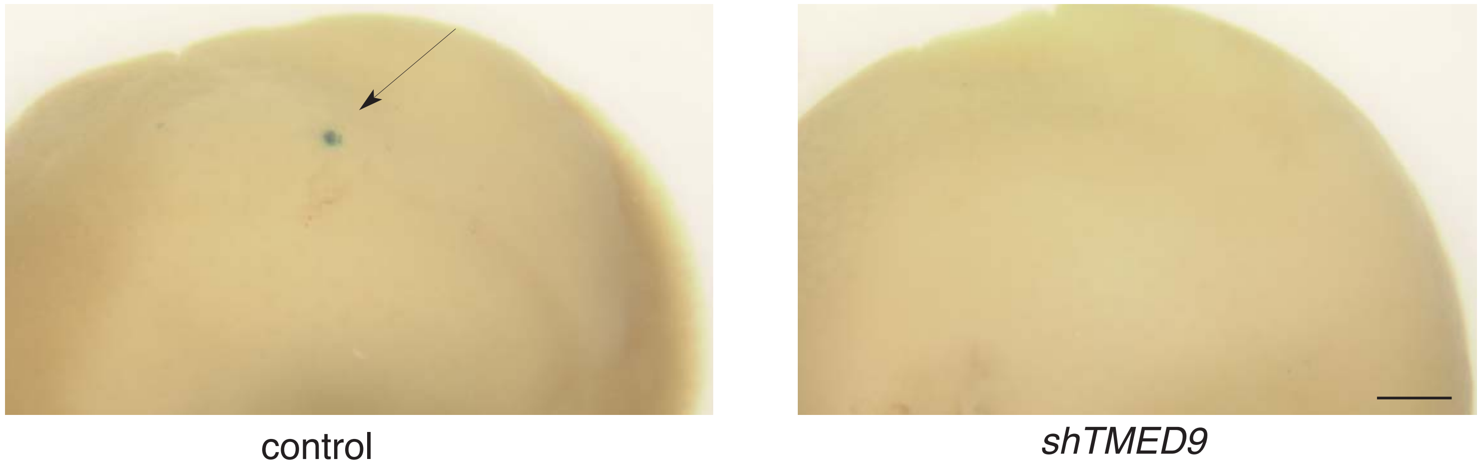

Mishra et al. Fig. S2

a RNA sequencing CC14^{shTMED9} vs. CC14^{control}

fold change up	Gene
13.7	C6orf15
9.4	WNT11
9.1	PXDN
9.1	UPK2
8.6	MUC16
8.4	AXL
7.0	AKAP12
6.2	VGLL1
6.1	CYMP
6.0	CXCL1
5.9	PLAU
5.9	PADI1
5.7	ELFN2
5.7	TNC
5.5	SOSTDC1
5.4	LOC101929723
4.9	VGLL3
4.8	FILIP1L
4.7	FN1
4.7	ADRB2
4.6	GPR87
4.5	WWTR1
4.4	CLIC5
4.4	MFAP2
4.2	PRSS2
4.0	FAM43A
3.9	CERCAM
3.9	NEURL3
3.9	ADGRF1
3.9	PHACTR3
3.9	ENPP5
3.8	CSF1
3.8	TACSTD2
3.7	KRT23
3.6	PHLDB2
3.6	SALL4
3.6	ALDH1A3
3.5	CXCL8
3.1	GALNT5
3.1	BZRAP1
3.0	FLJ12825
3.0	GPX8
3.0	ERP27
2.9	HERC6
2.9	FZD2
2.9	MATN3
2.9	QRICH2
2.9	HOXC9
2.9	SOCS3
2.8	C2orf54
2.8	LOC90246
2.8	SYTL3
2.8	LOXL4
2.8	RUNX2
2.8	ADPRH
2.7	SDPR
2.7	SLC2A12
2.7	FAM71F2
2.7	TNFRSF11B
2.7	FSCN1
2.7	PI3
2.6	UNC93A
2.6	CACNG4
2.6	TUBA1A
2.6	SEMA7A
2.6	POU6F1
2.6	PRSS23
2.5	ARL4C
2.5	MRAS
2.5	UCA1
2.5	KRT80
2.5	PGM2L1
2.5	EPHB2
2.5	RASL10B
2.5	CNN3
2.5	CD83
2.4	GOLGA7B
2.4	KRT17
2.4	LOXL3
2.4	TGFB1I1
2.4	TGFBI
2.4	DKK1
2.4	GYLTL1B
2.4	NDRG4
2.4	WNT3
2.4	APOLD1
2.4	CMTM3
2.4	GRHL3
2.4	BANK1
2.4	TSHZ2
2.3	BIRC3
2.3	SLC26A9
2.3	TMEM25
2.3	THSD4
2.3	ADRB1
2.3	SEMA3A
2.3	NEK11
2.3	CATSPER1
2.3	FKBP7
2.3	CEP112
2.3	ANXA6
2.3	MMP24
2.3	CYR61
2.3	PTGS2
2.3	CEACAM6
2.3	FRMD5
2.3	PRSS1
2.3	FER1L6
2.2	RGCC
2.2	STOX1
2.2	PLAC8
2.2	MACC1
2.2	ZNF358
2.2	C6orf222
2.2	ARHGDIB
2.2	HMGA2
2.2	KRT7
2.1	BDNF
2.1	CFH
2.1	SLC4A11
2.1	SCARA3
2.1	KLHL5
2.1	B3GNT7
2.1	LINC01605
2.1	GNA14
2.1	CNTNAP2
2.1	MMP7
2.1	P3H2
2.1	FAM198B
2.1	SPINK1
2.1	TGFB2
2.1	IL17RD
2.0	TMPRSS13
2.0	SEMA4C
2.0	NRP1
2.0	LGR5
2.0	CX3CL1
2.0	RHOB
2.0	RASSF10
2.0	TRAF1

fold change down	Gene
0.1	TMED9
0.2	MMP28
0.3	FAM129A
0.3	LAMP3
0.3	LOC344887
0.3	CSPG4
0.3	ADAM8
0.4	DPEP1
0.4	GPRIN3
0.4	ANXA13
0.4	ZDHHC8P1
0.4	ANXA10
0.4	MGAT3
0.4	AKR1B10
0.4	ADRA2A
0.4	ABCG1
0.4	COL9A3
0.4	PPP1R1B
0.4	LINC01612
0.4	TM4SF5
0.4	PRSS12
0.4	DQX1
0.4	CARNS1
0.4	KLF9
0.4	ARC
0.4	SLC5A1
0.5	CA9
0.5	HYAL1
0.5	PECR
0.5	NOXO1
0.5	CLDN2
0.5	TM4SF4
0.5	SNAI3
0.5	HR
0.5	P2RX6
0.5	ALDH3A1
0.5	KCNN4
0.5	CNIH4
0.5	CDC42EP5
0.5	GPT
0.5	PTGS1
0.5	C11orf24
0.5	LRRC66
0.5	ITPKA
0.5	PDZD3
0.5	GJB1
0.5	SMIM13
0.5	KLK11
0.5	FERMT3
0.5	SERPINA5
0.5	AKR1C1
0.5	AZGP1
0.5	CALU
0.5	MOGAT3
0.5	MYRFL
0.5	CDHR5
0.5	PDHA1
0.5	PIGA
0.5	SLC35D1

b

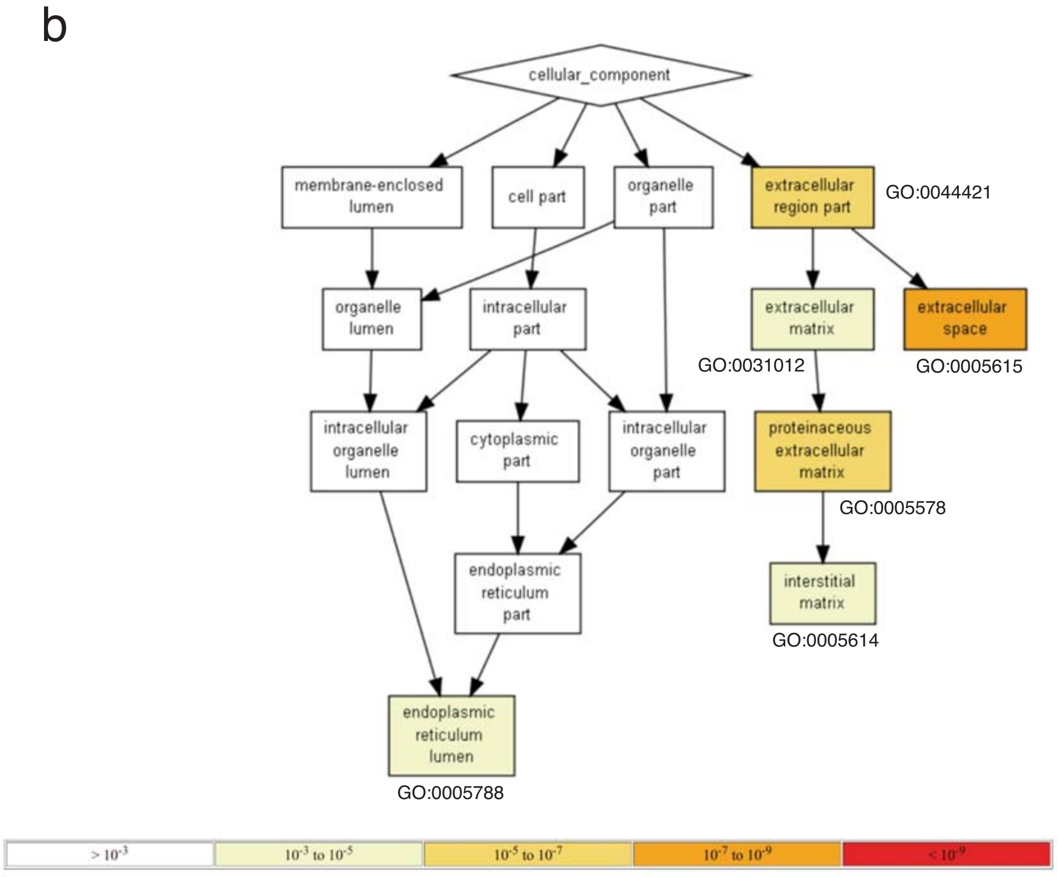

Mishra et al Fig S3

gene	FC	gene	FC	gene	FC	gene	FC
MIR30A	2282	SC5D	0.5	THPO	0.4	ZNF141	0.2
IFI44	14	SLC16A13	0.5	DDIT3	0.4	LINC01398	0.2
IFIT1	7.0	ATG4A	0.5	BLMH	0.4	RBPMS2	0.2
DDX60	5.2	HMGCS1	0.5	CAV1	0.4	NEUROG3	0.2
SOX8	4.2	DDIT4	0.5	LINC00261	0.4	IGFN1	0.2
CD74	4.1	MVK	0.5	ZNF83	0.4	GABRA3	0.2
IFIT2	4.1	IFITM10	0.5	ZNF585A	0.4	FAM129A	0.2
ALDH1A3	4.0	MIR22HG	0.5	CCT6B	0.4	C11orf86	0.2
SECTM1	3.9	MSMO1	0.5	TNNC1	0.4	ATOH8	0.2
CD14	3.7	LINC01011	0.5	TMEM86A	0.4	HEPACAM2	0.2
PLCB2	3.5	PFKFB4	0.5	TIGD3	0.4	RIMBP2	0.2
BTBD11	3.4	ZNF480	0.5	NMNAT3	0.4	ZNF461	0.2
ASCL2	3.3	CHST5	0.5	DNAJC12	0.4	VWA5B2	0.2
EEF1A2	3.1	ABHD4	0.5	C2orf48	0.4	CACNA2D2	0.1
CEMIP	3.1	ZFAND2A	0.5	ZNF358	0.4	MOCS1	0.1
FGF19	3.0	ZNF611	0.5	ZNF790	0.4	LCE1E	0.1
SERPINE1	3.0	ARMCX3	0.5	ZNF253	0.4	PAX4	0.1
IFIT3	2.9	ZNF675	0.5	GLI1	0.4	LINC01389	0.1
CTSS	2.8	TUSC8	0.5	ANK1	0.4	DACH1	0.1
CXCR4	2.7	ZNF577	0.5	ANXA13	0.4	TMED3	0.1
CNTD2	2.7	PPP1R3F	0.5	LPIN1	0.4	RASD2	0.1
FSCN1	2.7	TP53INP2	0.5	TOX2	0.4	EPHA4	0.1
CCNI2	2.7	MUC12	0.5	ZNF204P	0.4		
ZNF274	2.7	ZNF429	0.5	MAGEA3	0.4		
HIP1	2.7	PRSS16	0.5	IFFO1	0.4		
AKR1E2	2.7	SLC7A2	0.5	WBP5	0.4		
AQP3	2.7	PAG1	0.5	ZNF300	0.4		
KCNN4	2.6	GDF11	0.5	LOC441666	0.4		
PLIN2	2.6	ZNF613	0.5	ABCG1	0.4		
SH3BP5	2.6	KIAA1549L	0.5	LINC00665	0.4		
CYP24A1	2.6	DNAJB9	0.5	TFF3	0.4		
STEAP1	2.6	ZNF432	0.5	CXCL8	0.4		
PPP1R9A	2.6	CSAG1	0.5	ZNF529	0.4		
CLDN2	2.5	HERPUD1	0.5	GUSBP10	0.4		
HERC6	2.5	ATP8B2	0.5	FABP1	0.4		
CRABP2	2.5	GPC2	0.5	C6orf223	0.4		
USP2-AS1	2.4	ACSS2	0.5	ZNF571	0.4		
EPHB2	2.4	ZNF772	0.5	ZNF260	0.3		
BGN	2.4	ZNF566	0.5	ZNF345	0.3		
B3GNT7	2.4	TMPRSS13	0.5	NEURL1	0.3		
BIRC7	2.4	NUCB2	0.5	ZNF605	0.3		
RASAL1	2.4	QPRT	0.5	ATP11C	0.3		
ITPR1	2.3	ZNF567	0.5	KLK12	0.3		
NKD2	2.3	ZNF850	0.5	ZNF736	0.3		
KLHL3	2.3	CD99	0.5	ZSCAN12P1	0.3		
MUC1	2.2	REG4	0.5	SESN3	0.3		
CPNE7	2.2	PECAM1	0.5	GYG2	0.3		
SEMA3A	2.2	PLEKHG1	0.5	ZNF420	0.3		
ST6GAL1	2.2	RHOQ	0.5	ZNF615	0.3		
CEACAM1	2.2	CDKN1A	0.5	LINC01564	0.3		
GYLTL1B	2.2	ZNF585B	0.5	FFAR2	0.3		
VWA2	2.1	P3H2	0.5	IPO5P1	0.3		
YBX2	2.1	ZNF595	0.5	ZNF607	0.3		
ADAMTS17	2.1	NPPA-AS1	0.5	ZNF93	0.3		
MAPK12	2.1	WIPI1	0.5	RCOR2	0.3		
ROR1	2.1	ATOH1	0.5	DLL1	0.3		
B4GALNT1	2.1	CCDC163P	0.5	ATF7IP2	0.3		
RAMP1	2.1	ZNF350	0.4	IGFBP7	0.3		
CMTM3	2.0	SLC2A6	0.4	TMEM154	0.3		
CARD6	2.0	CCDC69	0.4	ADD2	0.3		
FN3K	2.0	SLC36A4	0.4	MAGEA6	0.3		
ADORA2B	2.0	MAGEA12	0.4	GUCA2A	0.3		
PLIN4	2.0	ENTPD8	0.4	FMOD	0.2		

Mishra et al. Fig.S4

*shTMED9*high;*shTMED3*low

shT9/shT3	gene	shT9/shT3	gene
14.1	*WNT11*	3.4	*FER1L6*
9.4	*ZNF461*	3.4	*SCARF2*
9.3	*CXCL8*	3.3	*THSD1*
7.5	*GABRA3*	3.3	*REN*
6.6	*LBH*	3.3	*ZNF833P*
6.5	*SCG5*	3.3	*ZNF117*
6.4	*TENM1*	3.3	*LTB*
6.3	*MRAS*	3.3	*36403*
6.3	*NNMT*	3.2	*FRZB*
6.2	*MVB12B*	3.2	*SLC2A6*
6.2	*ATF7IP2*	3.2	*SERP2*
6.2	*ZNF570*	3.2	*PROX1*
6.1	*ARHGAP31*	3.2	*ZNF569*
6.1	*DRP2*	3.1	*GPC2*
6.1	*IGFBP7*	3.1	*SATB2*
6.0	*FADS2*	3.1	*PPP2R3A*
5.9	*RAPSN*	3.0	*CNTNAP2*
5.9	*SIRPA*	3.0	*SNORD102*
5.8	*ZC4H2*	3.0	*LINC00930*
5.7	*PHLDB2*	3.0	*PPP1R3F*
5.4	*ZNF358*	3.0	*SUSD2*
5.3	*TSHZ2*	3.0	*KIAA1024*
5.2	*PCSK9*	3.0	*ELF5*
5.1	*ATL1*	3.0	*ZNF737*
5.0	*TOX*	2.9	*JAKMIP2*
4.9	*IL32*	2.9	*HSPA12A*
4.9	*SFMBT2*	2.9	*ABAT*
4.9	*ZNF704*	2.9	*ZNF75A*
4.8	*TNFRSF11B*	2.9	*LGR5*
4.7	*GYG2*	2.9	*CLCN4*
4.7	*GAL3ST4*	2.9	*BACH2*
4.7	*PI3*	2.8	*NAV1*
4.5	*P3H2*	2.8	*TP53INP1*
4.5	*IL23A*	2.7	*LINC01006*
4.5	*CAV1*	2.7	*LINC01252*
4.4	*FZD2*	2.7	*KIF3C*
4.4	*WFDC21P*	2.7	*SPTLC3*
4.3	*FSIP2*	2.6	*CD200*
4.3	*TMPRSS13*	2.6	*CBLN1*
4.2	*ZNF85*	2.5	*ZBTB10*
4.1	*ZNF529-AS1*	2.5	*LOC102723854*
4.1	*HDAC9*	2.5	*SBK1*
4.1	*LOXL4*	2.5	*ZNF701*
4.1	*TOX2*	2.5	*CLDN23*
4.0	*ANO3*	2.4	*ERV3-1*
3.9	*FGFBP1*	2.4	*ETS1*
3.9	*POLN*	2.3	*ANKRD29*
3.9	*RPS6KA2*	2.3	*CCL20*
3.9	*NDRG4*	2.3	*PDE5A*
3.8	*PLEKHG1*	2.3	*C2orf91*
3.8	*CCDC149*	2.2	*TMCC3*
3.7	*DPYSL3*	2.2	*TCEAL1*
3.6	*PREX1*	2.2	*PBX1*
3.6	*PTGS2*	2.2	*PALLD*
3.6	*CATSPER1*	2.2	*ZNF362*
3.6	*COL4A1*	2.2	*SCEL*
3.5	*RN7SL2*	2.1	*IGSF10*
3.5	*ZNF90*	2.1	*SNAI3-AS1*
3.5	*GRHL3*	2.1	*ZNF433*
3.4	*SLC7A8*	2.1	*SRR*
3.4	*TMEM217*		

shTMED9high;shTMED3high

gene
UPK2
AXL
PXDN
MUC16
AKAP12
PADI1
PLAU
GPR87
ADGRF1
ADRB2
CNN3
SLC26A9
TGFBI
MAPK12
CRABP2

*shTMED9*low;*shTMED3*low

gene
FABP1
CSPG4

Mishra et al. Fig.S5

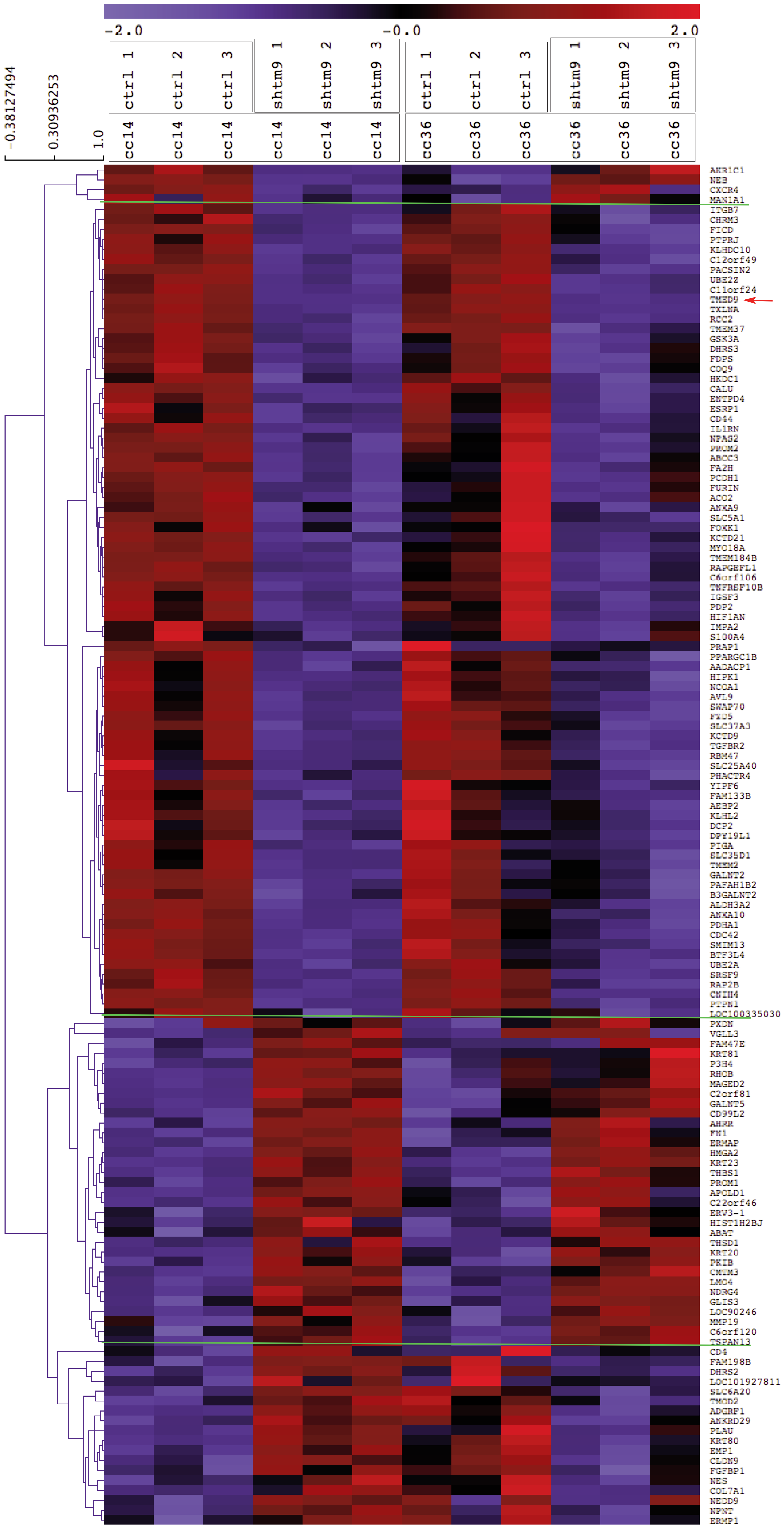

Mishra et al. Fig S6

a

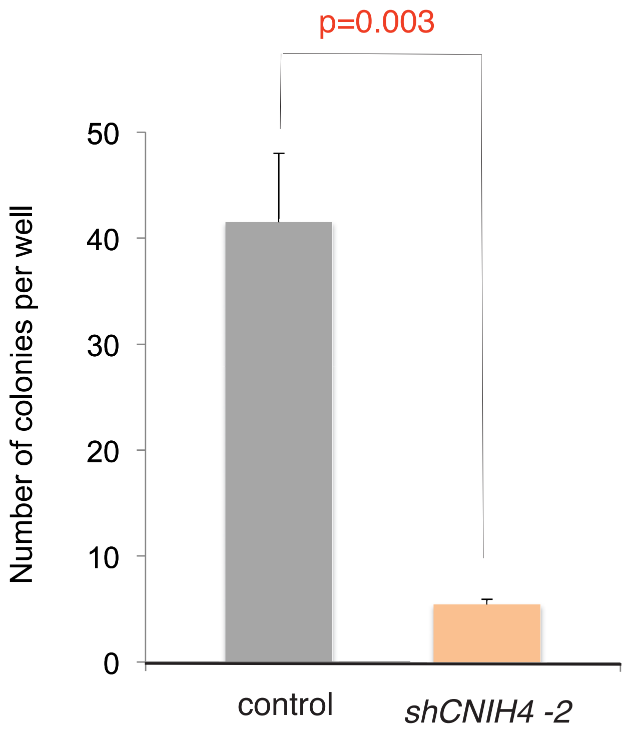

b

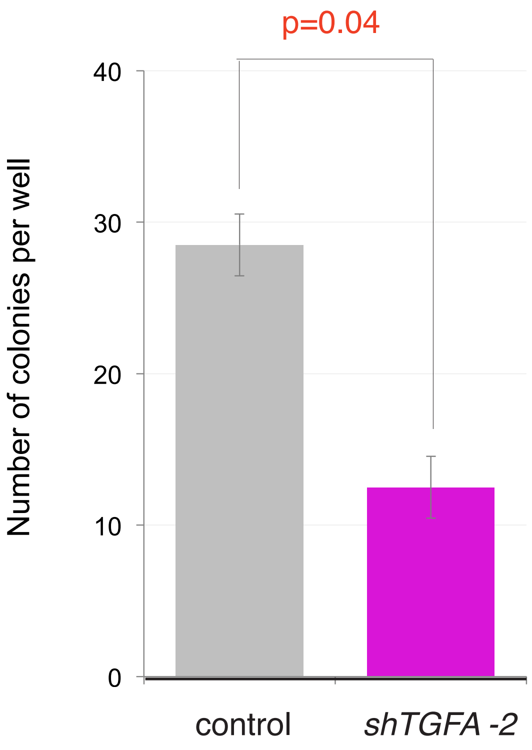

Mishra et al. Fig.S7

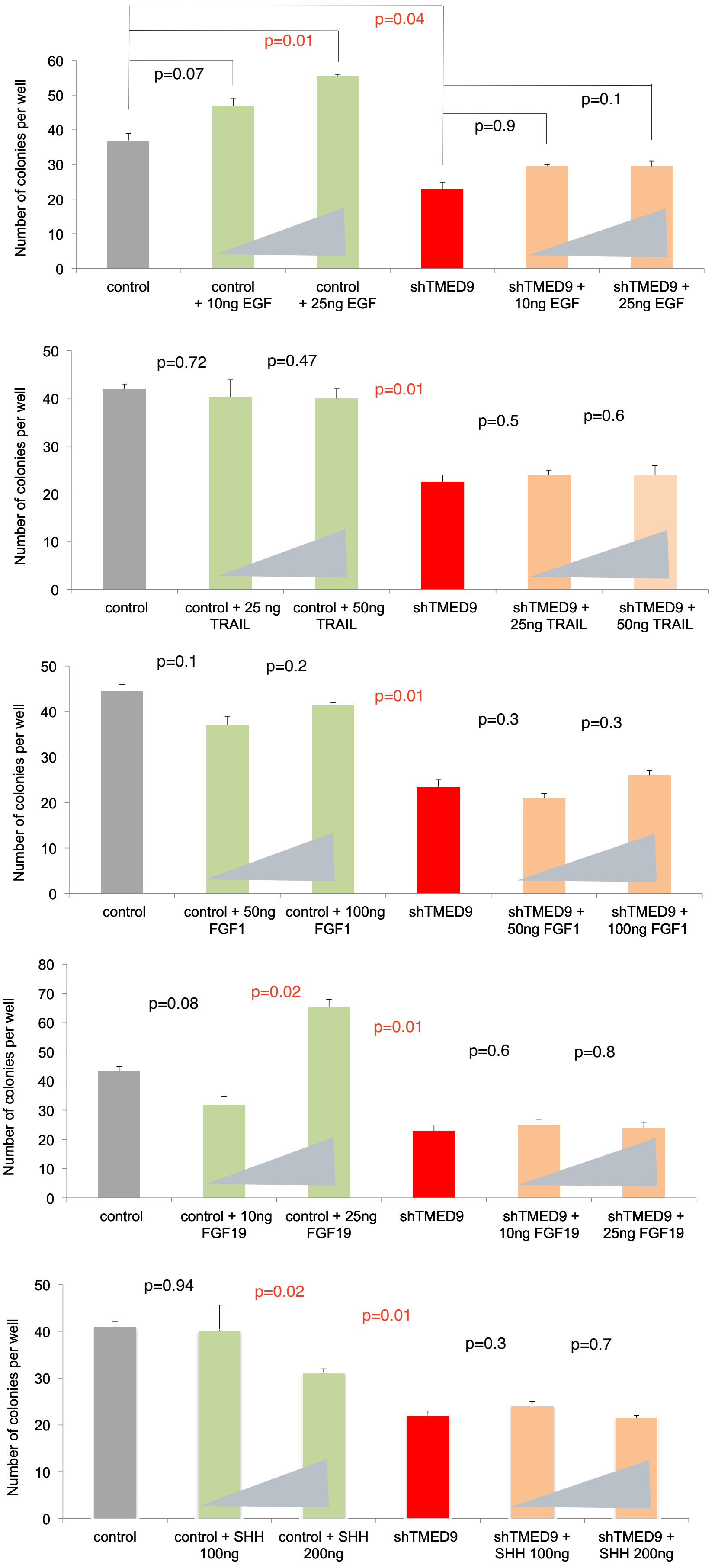

Mishra et al Fig. S8

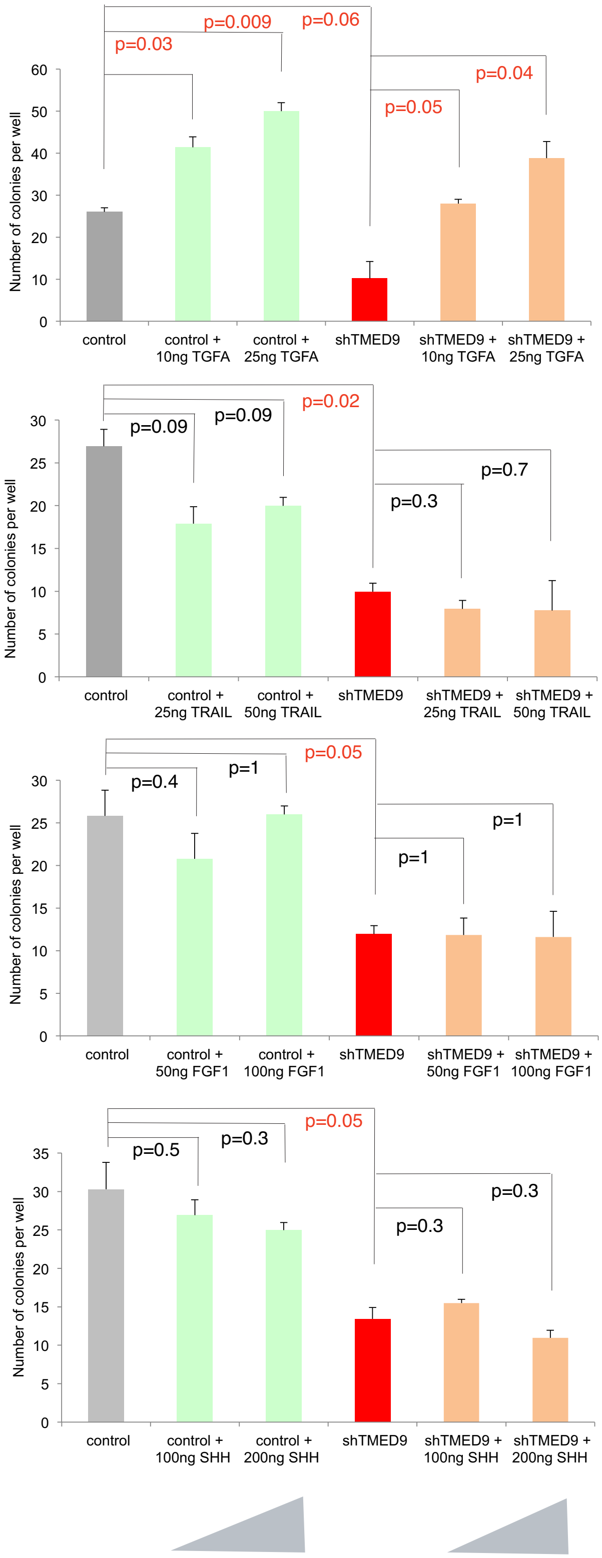

Mishra et al Fig. S9

a

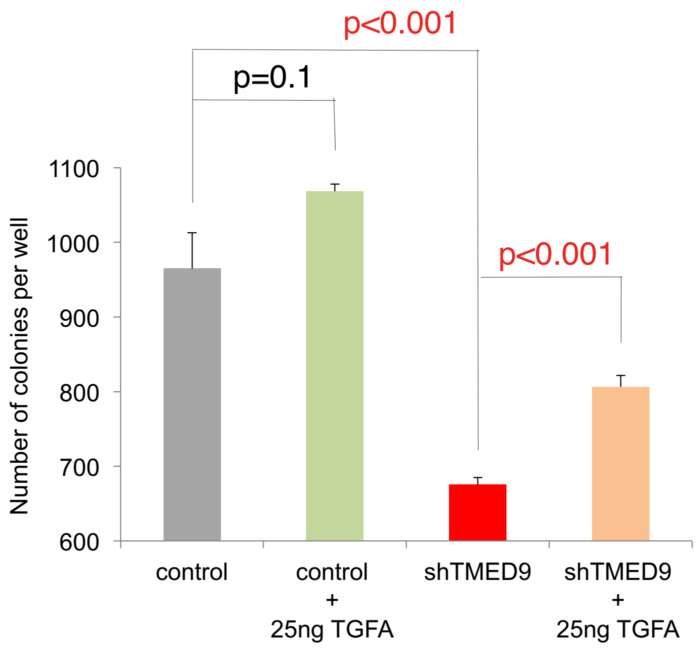

CC36

b

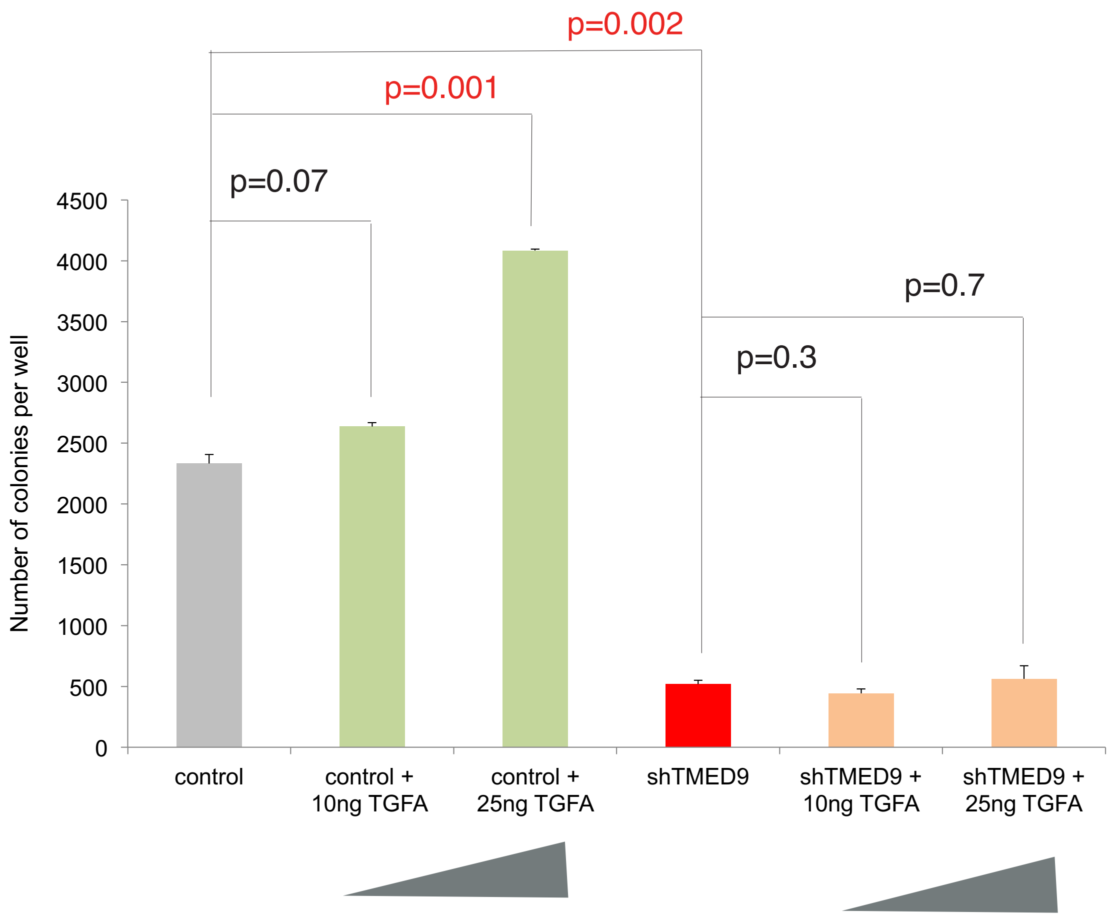

U251

Mishra et al Fig S10

EEA1 (early endosome) + TGFα

control *shTMED9*

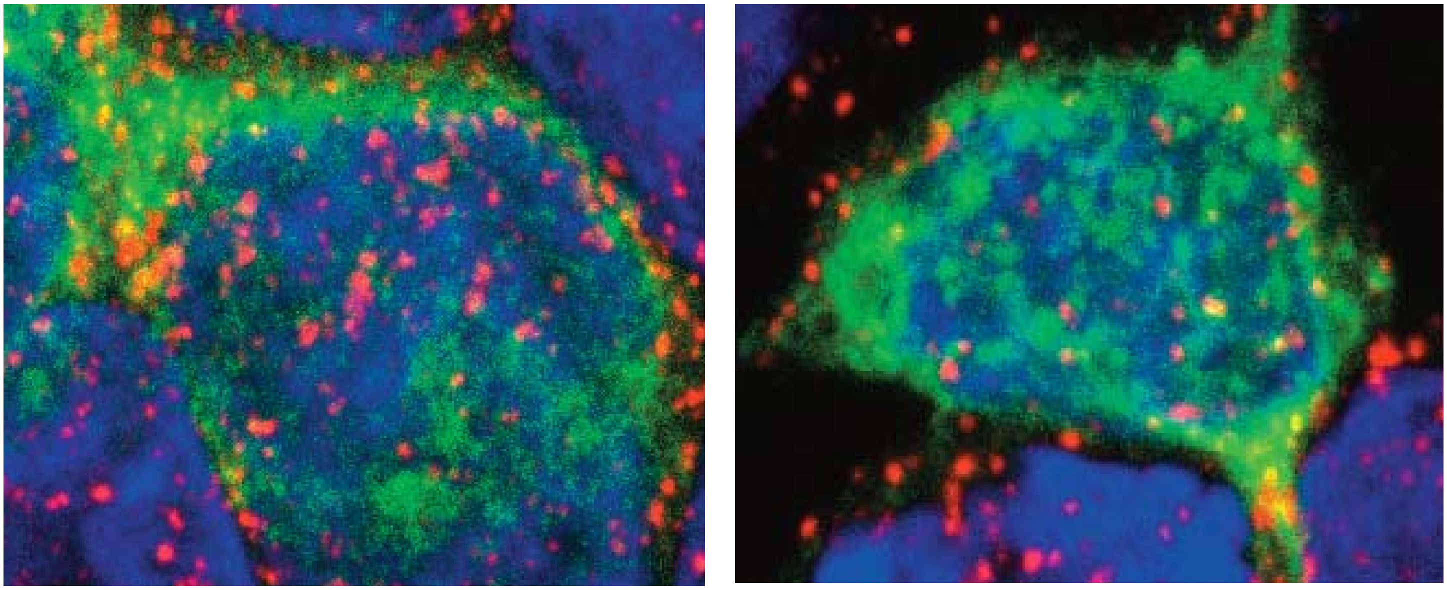

LAMP1 (lysosome) + TGFα

control *shTMED9*

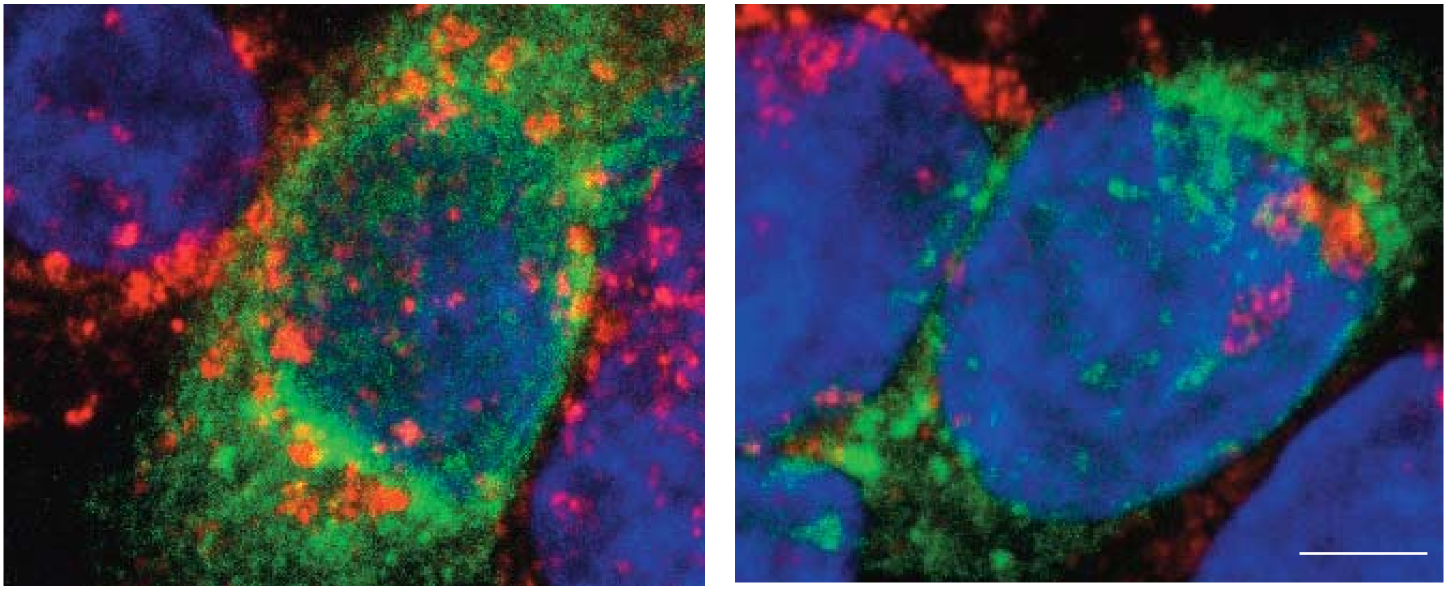

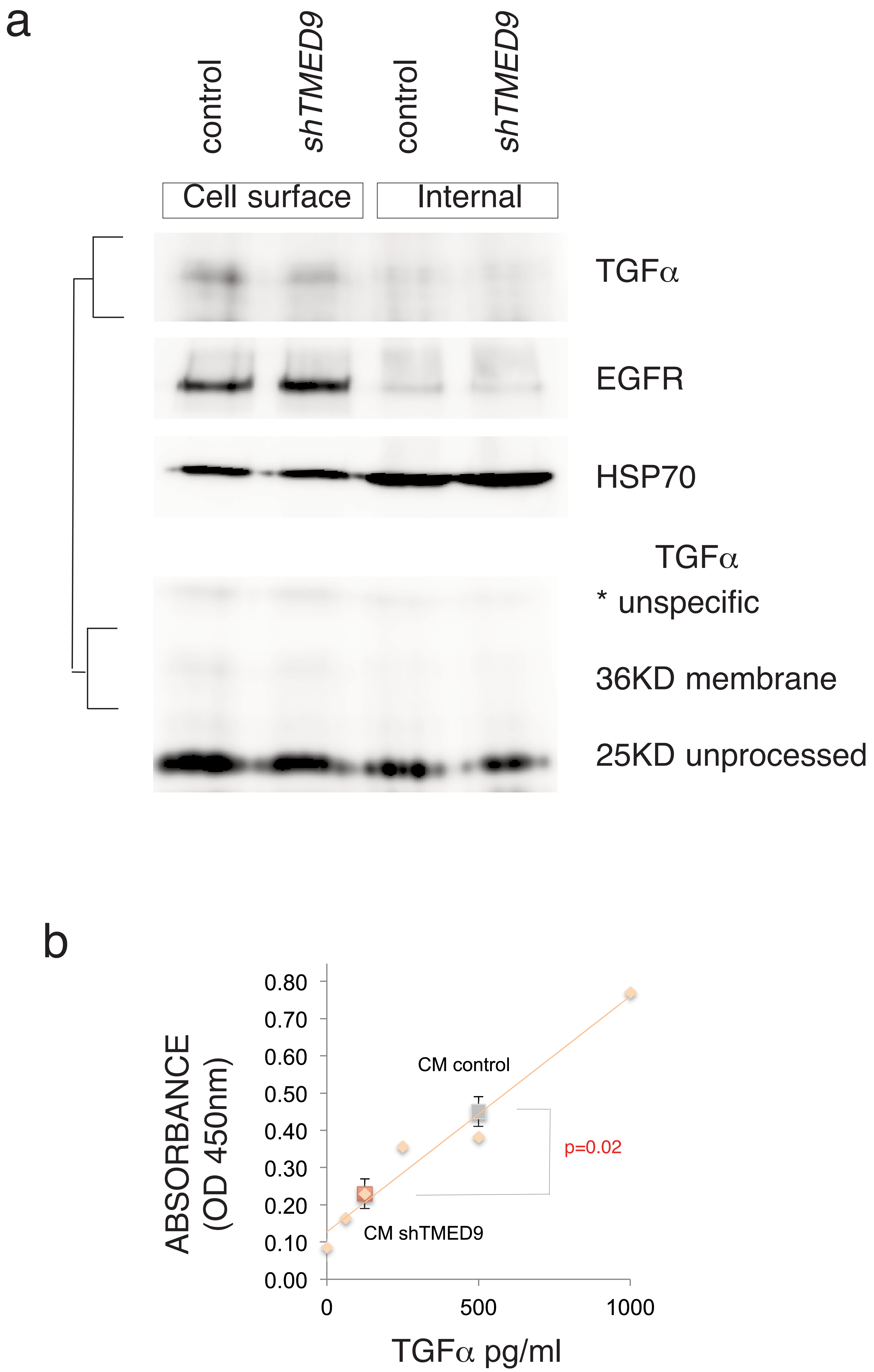

Mishra et al Fig S12

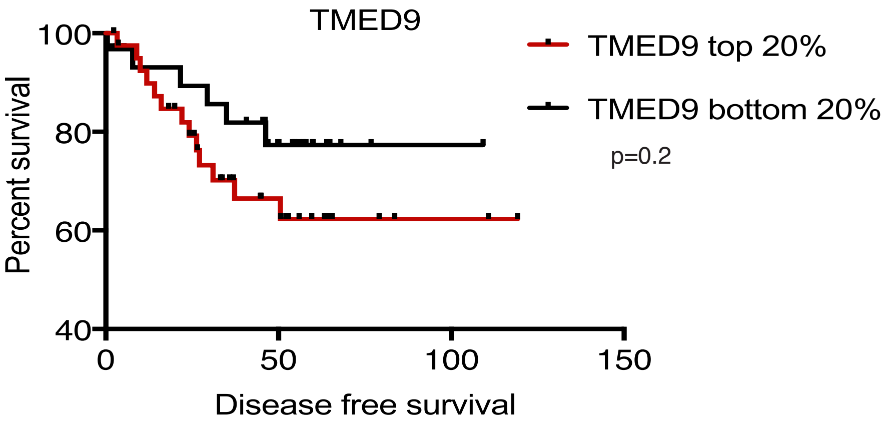

Mishra et al. Fig. S13

position (relative to TSS up to -5Kb)	GLI / TCF binding sites	FDR	strand
	TMED9		
-1075	GCCCTCCCA	0.17	-
-2070	GACCTCCCA	0.10	+
-4092	CACCTCCCA	0.14	-
-4958	GGCCTCCCA	0.17	+
	CNIH4		
329	CCTGGGTTGCCG	0.12	+
-1060	GACCCCCAA	0.12	-
-1647	CACCTCCCA	0.10	-
-1815	GACCTCCCA	0.10	-
-3326	GGCCTCCCA	0.12	+
-4086	GCCCACCCA	0.10	+
-4353	CACCACCCA	0.10	-
-4886	CCCCACCCA	0.12	+
	TGFA		
-813	GACCACCCA	0.06	-
	TMED3		
-2222	CAAGTTCAAAGGCC	0.06	-
-2797	TAACTTGAAAGAAA	0.20	-

Mishra et al. Fig. S14
